# Zbtb7a is a transducer for the control of promoter accessibility by NF-kappa B and multiple other transcription factors

**DOI:** 10.1371/journal.pbio.2004526

**Published:** 2018-05-29

**Authors:** José Miguel Ramos Pittol, Agata Oruba, Gerhard Mittler, Simona Saccani, Dominic van Essen

**Affiliations:** 1 Institute for Research on Cancer and Aging, Nice, Nice, France; 2 Max Planck Institute for Immunobiology and Epigenetics, Freiburg, Germany; University of California San Diego, United States of America

## Abstract

Gene expression in eukaryotes is controlled by DNA sequences at promoter and enhancer regions, whose accessibility for binding by regulatory proteins dictates their specific patterns of activity. Here, we identify the protein Zbtb7a as a factor required for inducible changes in accessibility driven by transcription factors (TFs). We show that Zbtb7a binds to a significant fraction of genomic promoters and enhancers, encompassing many target genes of nuclear factor kappa B (NFκB) p65 and a variety of other TFs. While Zbtb7a binding is not alone sufficient to directly activate promoters, it is required to enable TF-dependent control of accessibility and normal gene expression. Using p65 as a model TF, we show that Zbtb7a associates with promoters independently of client TF binding. Moreover, the presence of prebound Zbtb7a can specify promoters that are amenable to TF-induced changes in accessibility. Therefore, Zbtb7a represents a widely used promoter factor that transduces signals from other TFs to enable control of accessibility and regulation of gene expression.

## Author summary

Gene activation is driven by the binding of regulatory proteins to the specific DNA sequences that control each gene. However, these sequences are not always accessible for binding in every type of cell, and so differences in their accessibility can underlie the range of cell types in which particular genes can be activated. Although several cellular processes can alter the accessibilities of these sequences, it is still often unclear how these processes are directed to act at specific genes. We have discovered that the protein Zbtb7a binds near numerous gene-regulatory sequences throughout the genome and that it enables other DNA-binding proteins to trigger changes in their accessibility and to activate nearby genes. However, unlike many other factors that control gene activation, the binding of Zbtb7a alone does not seem to be sufficient to switch on gene expression; instead, its function is required for activation of genes that are independently bound by a specific set of transcription factors (TFs), and it could therefore be considered to “transduce” their gene-regulatory activities. The implication of this is that the presence or absence of Zbtb7a at any gene in a particular cell type may represent one of the aspects that can determine whether that gene is able to be activated or not.

## Introduction

The expression of protein-coding genes is governed by the combined activities of activating and repressing transcription factors (TFs), which bind in a specific fashion to regulatory sequences in proximal promoter regions and at distal enhancers. The abilities of TFs to bind to their genomic target sequences are limited by the chromatin context, or “accessibility,” at these sites. Accordingly, regulation of the accessibility of promoter and enhancer sequences represents one of the major, critical controls of the specificity of gene expression [1,2]. However, although many chromatin-remodeling complexes capable of regulating accessibility have been described [3,4], the upstream pathways that direct their activities to particular genomic loci and in response to defined signals are much less well characterised.

TFs differ in their requirements for binding to their target sites [5]. In many cases, TFs that are able to bind to less accessible regions can themselves trigger changes in local accessibility and thereby allow the recruitment of “secondary” TFs with more-stringent binding requirements, which can contribute to activation (or repression) of specific target genes [6,7]. This context-dependent mode of activation—which requires the presence of additional promoter/enhancer sequences that can be recognised and bound by secondary TFs—is well-established for the first-binding “pioneer” factors and has been described for other TFs, including NFκB [8,9].

The mechanistic steps underlying the activities of pioneer factors and similarly behaving TFs are still not well characterised [10]. An intriguing question is whether TFs with pioneer-like activity generally act by directly recruiting chromatin-modifying complexes to their binding sites or whether their activities may instead be transduced by preloaded factors at target promoters and enhancers. A corollary of the latter model is that the presence or absence of such “transducer” proteins could predetermine which sites are amenable to remodeling upon TF binding. However, so far, the identities or existence of possible transducers are unknown.

Separating regulation of accessibility from direct gene activation has been experimentally hard to approach because it is often difficult to distinguish whether changes in promoter accessibility represent downstream effects of the transcription process itself. However, we have previously shown that in mouse 3T3 fibroblasts, the principal activating NFκB subunit p65 is able to regulate target gene expression by 2 independent modes: either by direct transcriptional activation—dependent on its TA1 and TA2 domains, which interact with the Mediator complex—or indirectly, by controlling promoter accessibility for the binding of secondary TFs [9]. We term the as-yet undefined region(s) of p65 that mediate this latter activity as “TA3.” In this study, we have exploited this system as a model to identify molecular cofactors involved in accessibility-driven gene activation.

## Results

### 1. A SILAC-based screen identifies the protein Zbtb7a as a factor associated with context-dependent gene regulation

**Fig 1:**
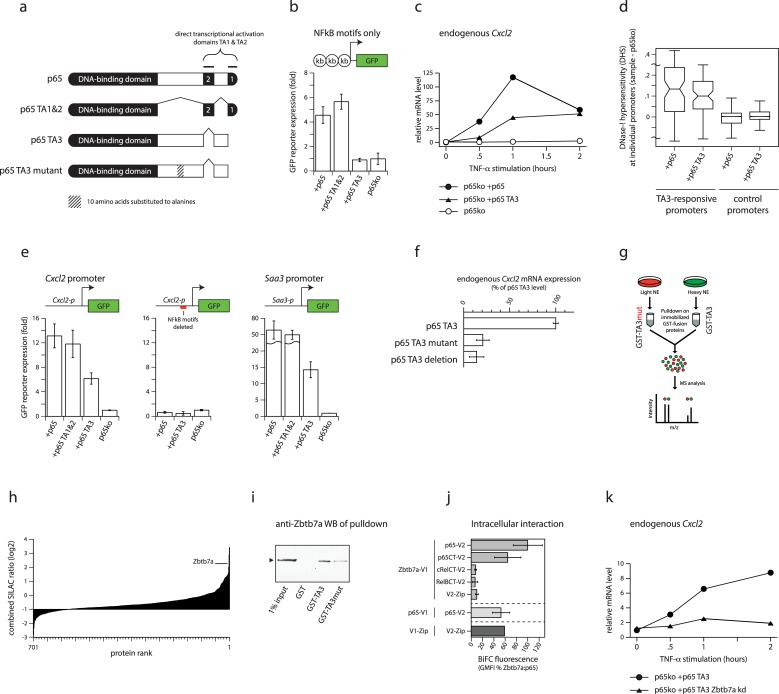
A SILAC-based screen identifies the protein Zbtb7a as a factor associated with context-dependent gene regulation. (A) Schematic illustration of p65 variants. (B) GFP reporter expression from a plasmid containing 3 tandem NFκB-binding motifs linked to a minimal promoter, in transfected p65-knockout fibroblasts expressing p65 variants and treated with TNF-α. Error bars indicate SEM. (C) Expression of endogenous *Cxcl2* mRNA in TNF-α-treated p65-knockout fibroblasts expressing p65 or p65 TA3, or in untransduced p65-knockout fibroblasts. mRNA levels are expressed relative to the level in unstimulated p65-knockout cells. (D) DNase-I hypersensitivity levels induced by p65 or by p65 TA3, at TA3-responsive or control (non-NFκB target) promoters in transduced p65-knockout fibroblasts. Induced levels represent differences in mean cut frequencies within ±600 bp surrounding the TSS, compared to p65-knockout fibroblasts. TA3-responsive promoters are defined as promoters of genes whose expression is activated by p65 TA3 and that are associated with p65 ChIP-seq peaks (see Materials and methods). Lines in boxplots indicate median values; whiskers extend to the most extreme data within 1.5× the IQR from the box; outliers are not shown. (E) GFP reporter expression from plasmids containing 1 kb promoter sequences from the TA3-responsive *Cxcl2* (left) and *Saa3* (right) genes, or the *Cxcl2* promoter with targeted mutations that delete the 2 consensus NFκB-binding motifs (centre), in transfected p65-knockout fibroblasts expressing p65 variants and treated with TNF-α. Error bars indicate SEM. (F) Expression of endogenous *Cxcl2* mRNA in TNF-α-treated p65-knockout fibroblasts expressing p65 variants (the TA3 deletion corresponds to removal of amino acids 361–396) (see S1E Fig). mRNA levels are expressed as the percentage of those in cells reconstituted with p65 TA3. Error bars indicate SEM. (G) Cartoon of the SILAC-based pull-down strategy to identify proteins that specifically interact with the functional TA3 region. (H) Combined log2 SILAC ratios of 701 proteins identified in GST-TA3 pull-down using NEs from TNF-α-treated HeLa cells. Ratios represent the means of heavy/light ratios derived from separate pull-downs with labels swapped between samples. (I) Validation of Zbtb7a as a functional TA3 interactor by pull-down from transfected HEK-293 cells overexpressing Zbtb7a, using GST alone, GST-TA3, or GST-TA3mut. Zbtb7a was detected by immunoblotting. Arrowhead indicates Zbtb7a. (J) Measurement of in vivo Zbtb7a interaction with p65 using BiFC. BiFC fluorescence values represent the GMFI of cells co-expressing the indicated proteins tagged with V1 (carboxy-terminal) or V2 (amino-terminal) fragments of Venus fluorescent protein, expressed as a percentage of the GMFI of cells expressing Zbtb7a-V1 plus p65-V2. Error bars indicate SEM. (K) Expression of endogenous *Cxcl2* mRNA in TNF-α-treated p65-knockout fibroblasts expressing p65 TA3, with or without shRNA knockdown of *Zbtb7a*. mRNA levels are expressed relative to the level in unstimulated cells without *Zbtb7a* knockdown. Statistical analysis of all experiments is provided as Supporting information, and numerical values underlying figures are reported in S1 Data. BiFC, bimolecular fluorescence complementation; ChIP-seq, chromatin immunoprecipitation sequencing; CT, carboxy terminal domain; GFP, green fluorescent protein; GMFI, geometric mean fluorescence intensity; GST, glutathione S-transferase; HEK-293, human embryonic kidney cells 293; IQR, interquartile range; m/z, mass to charge ratio; NE, nuclear extract; NFκB, nuclear factor kappa B; SEM, standard error of the mean; shRNA, short hairpin RNA; SILAC, stable isotope labelling of amino acids in cell culture; TNF-α, tumour necrosis factor alpha; TSS, transcription start site; Zip, control self-interacting leucine zipper sequence derived from yeast GCN4.

To uncouple direct transcriptional activation from the control of promoter accessibility, we generated a variant form of p65 lacking its 2 direct transcription activation domains. This variant (hereafter “p65 TA3”; Fig 1A) is completely unable to drive direct transcriptional activation of a reporter plasmid containing tandem NFκB-binding motifs linked to a minimal promoter (Fig 1B; in agreement with previous reports [11]). Despite its lack of direct activation capacity, p65 TA3 is able to robustly induce activation of the endogenous *Cxcl2* gene and other NFκB targets in 3T3 fibroblasts (hereafter simply “fibroblasts”) upon stimulation of the NFκB pathway by tumour necrosis factor alpha (TNF-α (Fig 1C and see later), albeit to lower magnitudes than those driven by the direct activation domains. Activation of endogenous promoters by p65 TA3 is associated with a strong induction of promoter accessibility, measured by increased DNase-I hypersensitivity (Fig 1D and S1A, S1B and S1C Fig).

These results suggest that the ability of p65 TA3 to induce transcription is “context dependent” and requires the presence of additional sequences within natural promoters, whose regulated accessibility could mediate gene activation. To investigate this, we generated reporter plasmids containing the natural approximately 1 kb promoter sequences from 2 p65 target genes, *Cxcl2* and *Saa3*. Episomal plasmids have been shown to be rapidly chromatinised upon transfection and can mimic normal patterns of accessibility at regulatory elements [12,13] (although precise nucleosome placement may differ from that at endogenous promoters [14,15]). For plasmids containing both promoters, p65 TA3 was able to trigger robust reporter gene activation (Fig 1E). Deletion of the NFκB-binding motifs within the cloned *Cxcl2* promoter abolished TA3-driven reporter expression, indicating that activation requires both direct binding of p65 TA3 as well as indirect regulation dependent on additional sequence elements present in natural promoters.

We identified the region within p65 TA3 responsible for its activity using a series of truncations, deletions, and substitution mutations (S1D, S1E and S1F Fig). The minimum active region (p65 amino acids 342–390) is not predicted to encode any known protein structural domains or catalytic activities and shares little primary sequence similarity to other non-NFκB proteins, but it is highly conserved among mammalian homologues of p65 (S1G, S1H and S1I Fig). The TA3 region displays conserved activity when assayed in either mouse or human cells, using the mouse *Cxcl2* promoter as a reporter (S1J Fig), demonstrating that the function (and potential interactor[s]) of the TA3 region is conserved between these species. Targeted mutations within the minimal TA3 region impaired its ability to induce activation of the endogenous *Cxcl2* promoter by more than 80% (Fig 1F, S1E Fig). Together, our results define the minimal TA3 region of p65 as a separable, conserved, functional element.

To search for factors that are generally required for TF-driven regulation of promoter accessibility and context-dependent gene activation, we used stable isotope labelling of amino acids in cell culture (SILAC) coupled with mass spectrometry (MS) to find proteins that could bind to the TA3 region but not to a functionally impaired mutant form (Fig 1G). We expressed both forms of TA3 as fusions to glutathione S-transferase (GST) in *Escherichia coli* and used these to enrich for binding proteins in nuclear extracts by affinity chromatography. Taking advantage of the conserved functionality of the TA3 region, we initially used nuclear extracts from TNF-α-stimulated human HeLa S3 cells as a source of potential interacting proteins. Among a small number of proteins with consistently high SILAC ratios (representing the ratio of binding to normal versus functionally impaired TA3), we identified the protein Zbtb7a (Fig 1H; empirical *P =* 0.0052; false discovery rate [FDR; Benjamini-Hochberg] = 0.0086), which has previously been reported as an interaction partner of p65 [16]. Zbtb7a exhibited the highest specificity for the TA3-containing bait when compared to pull-downs using GST alone (to identify likely promiscuously binding contaminants; S1K Fig); moreover, Zbtb7a also showed high SILAC ratios in pull-downs using mouse fibroblast nuclear extracts. In independent experiments, conventional (nonquantitative) MS analysis of proteins after fractionation by gel electrophoresis was consistently able to detect Zbtb7a-derived peptides in pull-downs using TA3, but with only reduced coverage when using the functionally impaired TA3 mutant (S1K Fig).

We validated the MS results by using the same GST fusion-protein baits to pull down a tagged form of Zbtb7a from transfected cells, followed by detection by western blotting (Fig 1I). Even under these conditions of strong overexpression—which promote low-affinity interactions—recovery of tagged Zbtb7a was still significantly reduced using the functionally impaired mutant of TA3, confirming the strong preference of the interaction for the functional form of TA3. The interaction between endogenous Zbtb7a and p65 proteins could be detected in unmodified fibroblasts by co-immunoprecipitation (S1I Fig).

Despite its high specificity for the functional TA3 region, Zbtb7a-derived peptides were detected by conventional MS with lower coverage than those of several other proteins that interact with the full C-terminus of p65, suggesting that the interaction between TA3 and Zbtb7a may be less structurally robust under the biochemical conditions used. Therefore, to confirm the intracellular interaction of Zbtb7a and the p65 C-terminus, independently of any biochemical extraction conditions, we used bimolecular fluorescence complementation (BiFC). We expressed Zbtb7a as a fusion protein with the N-terminal fragment of the fluorescent protein Venus (V1), together with p65 fused to the Venus C-terminus (V2). Cells expressing fusion proteins individually were nonfluorescent, and only background fluorescence could be detected from cells co-expressing Zbtb7a-V1 together with control protein fragments fused to V2. In contrast, co-expression of Zbtb7a and p65 fused to complementary Venus fragments yielded strong nuclear fluorescence, demonstrating their intracellular interaction (Fig 1J). Interaction of Zbtb7a could also be detected with the isolated C-terminus of p65 (Fig 1J) or with the TA3 region alone (S1M Fig), but not with the unrelated C-termini of other NFκB subunits, cRel and RelB. To address whether the interaction with the p65 TA3 region requires co-assembly with other cellular or nuclear factors, we expressed Zbtb7a fragments in vitro using a cell-free linked transcription/translation system. We could detect a reliable binding of the zinc-finger domain of Zbtb7a to GST-TA3 and not to GST alone (even using higher bait protein amounts; S1N Fig), suggesting that the interaction is likely to be direct and independent of other specific co-interacting proteins (although we cannot rule out the participation of chaperones or other generic factors that may be present in the cell-free expression system).

Knockdown of Zbtb7a using short hairpin RNA (shRNA; described later) abolished the ability of p65 TA3 to induce expression of the endogenous *Cxcl2* gene in response to TNF-α stimulation (Fig 1K) but only partially impaired its activation by full-length p65 (S1O Fig), indicating that Zbtb7a is functionally required for TA3 activity.

From these experiments, Zbtb7a emerges as a protein that interacts specifically with the functional form of the TA3 region of p65 and that is required for its context-dependent transcriptional activation. It is therefore a strong candidate as a cofactor that may be mechanistically involved in TF-dependent regulation of target promoter accessibility.

### 2. Zbtb7a is a widespread promoter- and enhancer-associated factor

**Fig 2:**
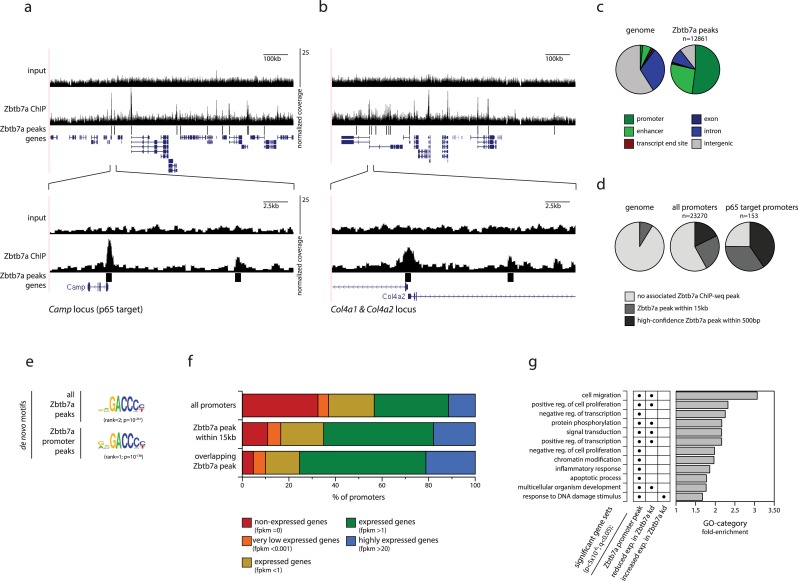
Zbtb7a is a widespread promoter- and enhancer-associated factor. (A, B) Genome browser example tracks of a Zbtb7a ChIP-seq and input coverage (top) across representative 1 Mb genomic intervals, illustrating the prominent overlap between Zbtb7a peaks and TSSs, and (bottom) zoomed-in on 25 kb regions including the Zbtb7a-bound *Camp* (A) and *Col4a1/2* (B) gene promoters. Lower tracks indicate predicted Zbtb7a binding peaks and RefSeq genes. (C) Fractions of the genome (left pie) and of Zbtb7a ChIP-seq peaks (right pie) that overlap with selected genomic features. (D) Fractions of the genome, of all promoters, and of p65 target promoters that are associated with predicted Zbtb7a ChIP-seq peaks. (E) DNA sequence motifs matching the described binding specificity of Zbtb7a, enriched among all (top) and promoter-associated (bottom) Zbtb7a ChIP-seq peaks, identified by de novo motif prediction. (F) Fractions of all promoters (top) or of promoters overlapping (bottom) or within 15 kb of (middle) predicted Zbtb7a ChIP-seq peaks that are expressed in fibroblasts at the indicated mRNA levels. (G) Enrichment of GO annotations among genes whose promoters are associated with Zbtb7a peaks (first column, “Zbtb7a promoter peak”) or with increased or decreased mRNA expression in control fibroblasts, compared to Zbtb7a-knockdown fibroblasts (second and third columns). Dots indicate statistically enriched annotations (*P <* 5 × 10^−6^; corrected q < 0.05). The most-significantly enriched annotations among genes with Zbtb7a-associated promoters are shown, of which some are also enriched among genes exhibiting Zbtb7a-regulated expression. Bars indicate levels of enrichment compared to all annotated genes. Statistical analysis is provided as Supporting information, and numerical values underlying figures are reported in S1 Data. ChIP-seq, chromatin immunoprecipitation sequencing; FPKM, RNA-sequencing fragments per kilobase transcript per million reads; GO, gene ontology; RefSeq, NCBI reference sequence database; TSS, transcription start site.

Zbtb7a (also named FBI-1, LRF, or Pokemon) belongs to the POZ-ZF family of proteins—characterised by linked POZ/BTB (pox virus zinc finger/broad-complex, tramtrack, bric-a-brac) and zinc-finger (ZF) domains—and has been previously reported to associate with promoters of several well-studied model genes (reviewed in [17]). To examine the binding specificity of Zbtb7a at the genome-wide scale, we performed chromatin immunoprecipitation sequencing (ChIP-seq) for Zbtb7a in fibroblasts. Enrichment of the Zbtb7a ChIP signal at predicted peaks was highly reproducible between independent replicate experiments, although the magnitude of the detected signal at many peaks was moderate. We could robustly detect Zbtb7a binding at thousands of genomic loci (12,861 predicted peaks; Fig 2A, 2B, 2C and 2D), which predominantly represent promoter and enhancer regions (Fig 2C). Consistent with a role in regulating p65 function, Zbtb7a is strongly enriched at p65 target promoters (Fig 2D, S2A, S2B, S2C and S2D Fig) and at p65-bound intergenic peaks (S2E and S2F Fig), and Zbtb7a is among the top-ranking factors associated with p65-bound promoters among 109 distinct genome-wide ChIP-seq datasets (S2G Fig). However, Zbtb7a is not restricted to NFκB target promoters, and indeed, a large fraction of all genomic promoters are associated with overlapping—or nearby—Zbtb7a binding (up to 17% or 41%, respectively, of all promoters in fibroblasts; Fig 2D), with a preference for guanine-cytosine (GC)-rich and CpG island (CGI)-containing promoters (S2H and S2I Fig). Thus, Zbtb7a binding represents a very prevalent event, occurring at many promoters and suggesting a widespread mechanistic role in gene regulation.

De novo motif discovery at Zbtb7a-bound ChIP-seq peaks and promoters recovered the previously described Zbtb7a binding motif, confirming the ChIP specificity ([18,19]; Fig 2E, S2J Fig). The Zbtb7a motif can be detected at up to 66% of Zbtb7a peaks, indicating that Zbtb7a binding is generally a specific, sequence-encoded feature rather than an indirect association with transcriptionally active or GC-rich genomic regions, and also revealing that Zbtb7a binding is hard-wired into many promoter and enhancer sequences.

The large number of promoters bound by Zbtb7a is much higher than those bound by many conventional TFs and is more comparable to known transcriptional cofactors (S2K Fig). Zbtb7a-bound promoters are enriched for those of expressed genes but also include those of many nontranscribed genes and of genes with very low expression levels (Fig 2F). This is not a consequence of different levels of Zbtb7a occupancy because the magnitudes of the Zbtb7a ChIP signal at bound promoters with different expression levels are comparable (S2L Fig). Therefore, consistent with a role as a regulatory cofactor, Zbtb7a binding is not alone sufficient to autonomously activate gene expression.

To identify pathways and processes associated with possible regulation by Zbtb7a, we examined gene ontology (GO) annotations of genes with Zbtb7a-bound promoters. Zbtb7a target promoters displayed highly significant enrichments for multiple GO terms (Fig 2G), prominently including processes linked to cell proliferation and apoptosis, gene regulation, and signal transduction (including inflammatory and innate immune responses, which encompass many of the NFκB target promoters, as well as several specific developmental signalling pathways [S2M Fig]). These data support the notion that the involvement of Zbtb7a in gene regulation is a feature shared by multiple pathways (in addition to that of NFκB).

### 3. Zbtb7a is required for ongoing regulation of accessibility at a subset of its genomic binding sites

We used experimental depletion of Zbtb7a coupled with quantitative genome-wide DNase-I hypersensitivity mapping to examine the involvement of Zbtb7a in regulating promoter and enhancer accessibility.

We depleted Zbtb7a in fibroblasts using stable shRNA (S3A, S3B, S3C, S3D and S3E Fig). In agreement with previous studies [20], we found that even incomplete depletion of Zbtb7a (to roughly 20%–30% of normal protein levels; S3E Fig) resulted in a gradual impairment in cell viability and proliferation. Therefore, to minimise indirect effects, we avoided long-term culture of *Zbtb7a*-knockdown cells and performed all analyses within approximately 5 to 10 passages. For similar reasons, we also felt that (for these experiments) the knockdown approach is preferable to analysing cell lines from Zbtb7a-knockout mice because it avoids any indirect effects that may arise from the different developmental histories of distinct, independently derived cell lines.

**Fig 3:**
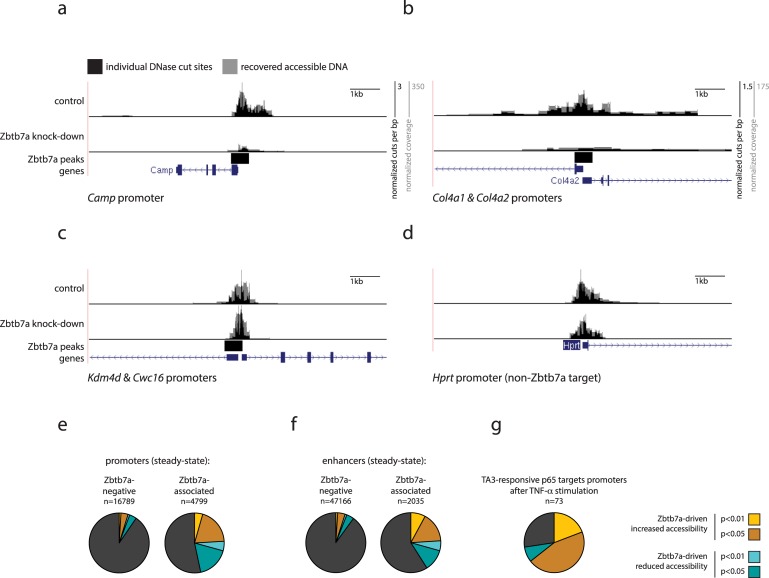
Zbtb7a is required for ongoing regulation of accessibility at a subset of its genomic binding sites. (A–D) Genome browser example tracks of DNase-I hypersensitivity surrounding (A, B) the *Camp* and *Col4a1/2* gene promoters (Zbtb7a-bound and Zbtb7a-dependent accessibility), (C) the promoter region of the *JmjD2d* and *Cwc15* genes (Zbtb7a-bound but ongoing accessibility is Zbtb7a-independent), and (D) the control *Hprt* promoter (non Zbtb7a-bound), in control or Zbtb7a-knockdown fibroblasts. Lower tracks indicate predicted Zbtb7a binding peaks and RefSeq genes. (E–G) Fractions of (E) promoters, (F) enhancers, and (G) TA3-responsive p65 target promoters that exhibit significant evidence for Zbtb7a-regulated accessibility in fibroblasts. Left (E, F): regions without any associated Zbtb7a peak (“Zbtb7a-negative”); right (E, F): regions with associated Zbtb7a peaks. Yellow/cyan slices indicate promoters with increased/decreased DNase-I hypersensitivity in control fibroblasts compared to Zbtb7a-knockdown fibroblasts, at the indicated *P* value cutoffs. Statistical analysis is provided as Supporting information, and numerical values underlying figures are reported in S1 Data. RefSeq, NCBI reference sequence database; TNF-α, tumour necrosis factor alpha.

Using DNase-I hypersensitivity mapping, we could detect altered levels of accessibility at a significant fraction of Zbtb7a-associated promoters and enhancers upon knockdown of Zbtb7a (Fig 3A, 3B, 3E, 3F and S3F, S3G, S3H, S3I, S3J and S3K Fig). Accessibility was largely unchanged at Zbtb7a-unbound regions (Fig 3D, 3E and 3F), indicating that this is a direct effect mediated by Zbtb7a and ruling out a nonspecific role for Zbtb7a in controlling overall chromatin structure. Therefore, Zbtb7a is necessary for ongoing regulation of accessibility at many of its binding sites. Regions that exhibit Zbtb7a-dependent changes in accessibility include around 47% of Zbtb7a-bound promoters (or 9% of all genomic promoters) and comprise roughly equal proportions of Zbtb7a-dependent increased and decreased accessibility (Fig 3E and 3F), implying that additional factors or signals may determine the outcome of Zbtb7a-dependent regulation. Notably, at TA3-responsive p65 target promoters, regulation through Zbtb7a almost exclusively mediates increased promoter accessibility (Fig 3G), suggesting that particular biological pathways may predominantly utilise Zbtb7a in a uniform manner.

To determine whether Zbtb7a-dependent increases in promoter or enhancer accessibility act to enable binding of TFs, we analysed DNase-I footprints at known TF binding motifs within Zbtb7a-regulated regions. We first analysed the recognition motifs of known TFs to identify those that are enriched among the complete set of promoters and enhancers that are active in fibroblasts. Although the identities of the specific TFs that bind to each enriched motif are often uncertain (since many motifs may be bound by more than one known or unknown TF), these motifs nonetheless reflect targets of TFs that are relevant for gene regulation in fibroblasts. Many of these motifs are also enriched within the subset of regions exhibiting Zbtb7a-dependent accessibility, suggesting that these regions indeed include functional binding sites for TFs. We next mapped DNase-I cut sites across instances of each motif within regions exhibiting Zbtb7a-dependent accessibility: for many motifs, a clear and significant “footprint” of differential DNase-I sensitivity is apparent at these sequences, indicative of direct protein-DNA binding ([21,22]; S3L Fig). Thus, Zbtb7a-dependent changes to the accessibility of promoters and enhancers expose binding motifs that can be recognised and bound by secondary TFs. Finally, we directly tested the requirement for Zbtb7a for motif-binding by TFs, by analysing the magnitudes of motif footprints in *Zbtb7a*-knockdown cells. Indeed, for many motifs that are present within Zbtb7a-regulated regions, binding footprints are markedly reduced upon Zbtb7a depletion, indicative of Zbtb7a-dependent TF occupancy (S3M Fig). Consistent with this, Zbtb7a is positioned as one of the most upstream-acting factors in previous network analyses of TF binding [23].

To verify whether Zbtb7a-depenedent regulation of accessibility is required for p65-driven recruitment of specific secondary TFs to NFκB target promoters, we performed ChIP for 2 previously characterised factors, Cebpb and JunD [9]. In both cases, promoter-binding requires stimulation of the NFκB pathway by TNF-α as well as the activity of p65 (S3N and S3O Fig). However, we find that binding to multiple NFκB target promoters is significantly impaired or even abolished upon knockdown of Zbtb7a, confirming that Zbtb7a is required for p65-driven regulation of secondary TF recruitment.

Despite the Zbtb7a-dependent regulation of accessibility at many promoters and enhancers, however, a major fraction of Zbtb7a-bound sites do not exhibit any detectable requirement for Zbtb7a for steady-state accessibility (Fig 3C, 3E and 3F, S3H and S3K Fig; see also section 6 later). These sites include promoters of both nonexpressed and expressed genes and reveal that the simple presence of Zbtb7a is not itself sufficient to trigger changes in accessibility at all sites.

### 4. Target genes of a diverse set of TFs exhibit Zbtb7a-dependent promoter accessibility and expression

The findings above suggest a model in which promoter- and enhancer-bound Zbtb7a acts as a general cofactor, which is required—but not sufficient—for regulation of local accessibility and gene activation. Moreover, the enrichment of Zbtb7a-bound promoters for a variety of biological processes hints that Zbtb7a may be utilised by a diverse set of TFs, in addition to NFκB p65.

**Fig 4:**
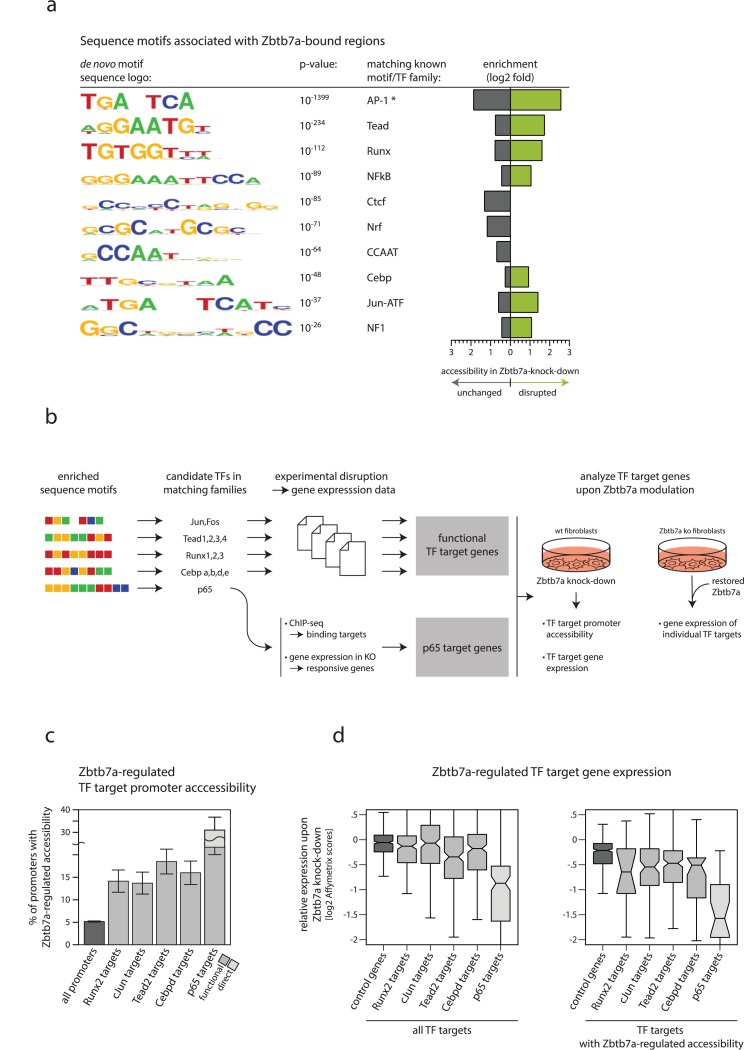
Target genes of a diverse set of TFs exhibit Zbtb7a-dependent promoter accessibility and expression. (A) DNA sequence motifs that are consistently enriched at Zbtb7a ChIP-seq peaks (note that the binding motif for Zbtb7a itself is also highly enriched, but not shown here). Known TF families with specificities matching each motif are indicated. Grey and green bars display the log2 fold-enrichment of each motif among the subset of peaks exhibiting unchanged (grey) or disrupted (green) DNase-I hypersensitivity levels in *Zbtb7a*-knockdown fibroblasts. *Note that the motif TGANTCA belongs to a previously identified set of motifs that are commonly found to be enriched within unrelated genome-wide datasets [24], suggesting caution in interpretation based on motif enrichment alone, in this instance. (B) Schematic illustration of the strategy for identifying candidate Zbtb7a-utilising TF target genes and for experimentally analysing Zbtb7a dependence of promoter accessibility and gene expression. (C) Percentages of all promoters, and of target promoters of selected TFs, that exhibit Zbtb7a-regulated accessibility (experimentally defined as significantly reduced DNase-I hypersensitivity in Zbtb7a-knockdown fibroblasts, using a statistical cutoff of *P <* 0.05). Candidate TFs were identified using the scheme in panel b, and only targets of individual TFs that exhibit enrichment for Zbtb7a-regulated accessibility are shown. Error bars indicate 95% CIs. Significance of enrichments: Runx2 q = 1.4 × 10^−6^; cJun *P =* 1.4 × 10^−6^; Tead2 q = 2.3 × 10^−11^; Cebpd q = 1.7 × 10^−8^; p65 *P =* 1.9 × 10^−29^. (D) Microarray-based analysis of changes in mRNA expression levels upon Zbtb7a knockdown in fibroblasts, among target genes of selected TFs. Left: all TF target genes; right: target genes that also exhibit Zbtb7a-regulated accessibility. Lines in boxplots indicate median values; whiskers extend to the most extreme data within 1.5× the IQR from the box; outliers are not shown. Significance of expression differences: Runx2 *P =* 8.9 × 10^−3^; cJun *P =* 2.3 × 10^−7^; Tead2 *P =* 9.7 × 10^−11^; Cebpd *P =* 4.9 × 10^−6^; p65 *P =* 1.8 × 10^−14^. Additional details of statistical analysis are provided as Supporting information, and numerical values underlying figures are reported in S1 Data. ChIP-seq, chromatin immunoprecipitation sequencing; IQR, interquartile range; TF, transcription factor.

With this in mind, we set out to identify examples of other TFs that may exploit or utilise promoter-associated Zbtb7a in fibroblasts. As a first step, we used de novo motif prediction to search in an unbiased fashion for DNA sequences that are overrepresented at or nearby Zbtb7a-bound promoters or peaks, irrespective of their measured levels of steady-state accessibility regulation by Zbtb7a. Motif prediction was consistent across several matched background sets, including total and CGI promoters, ruling out that enrichment could be biased by the strong overlap of Zbtb7a with promoter regions. We consistently found the motif matching the consensus NFκB binding site (Fig 4A), confirming that this is a feasible approach to reveal the sequence specificities of candidate Zbtb7a-utilising TFs. In addition, we identified several other sequence motifs that are significantly overrepresented at Zbtb7a-associated peaks and promoters. Notably, a number of the most significant de novo motifs closely resembled the consensus binding sequences for known TF families (indicated in Fig 4A), including several involved in developmental and signal-dependent gene activation. We therefore addressed the possibility that one or more TFs recognising each enriched motif may utilise Zbtb7a as a promoter-bound adaptor protein for the regulation of accessibility and/or gene activation, analogously to the TA3 region of p65.

We considered motifs that also displayed preferential enrichment at sites with disrupted accessibility in Zbtb7a-knockdown cells, consistent with a functional link to Zbtb7a in fibroblasts under steady-state conditions (Fig 4A). Following the strategy illustrated in Fig 4B, starting from each de novo motif, we identified candidate TFs with matching known DNA-binding specificities (belonging to the AP1, Tead, Runx, NFκB, Cepb, and NF1 families) and assembled experimentally defined lists of functional target genes using publicly available gene expression datasets generated in mouse fibroblasts. In addition, we constructed a high-confidence list of putative direct targets of NFκB p65 by performing ChIP-seq for p65 and combining this with microarray gene expression analysis in normal and p65-knockout fibroblasts. Finally, for each candidate TF, we analysed changes in target promoter accessibility and target gene expression upon experimental depletion of Zbtb7a, both by genome-wide analyses in Zbtb7a-knockdown fibroblasts and by analysis of individual TF target gene expression by Zbtb7a-knockout cells after experimental restoration of Zbtb7a (Fig 4B).

Using this approach, we were able to identify several TFs belonging to distinct families whose target gene promoters exhibit significantly impaired accessibility in Zbtb7a-knockdown cells (Fig 4C). The fraction of TF target promoters with strong evidence for Zbtb7a-dependent regulation of accessibility ranged from 14% (c-Jun) to 21% (p65), suggesting that Zbtb7a may be utilised at only a subset of TF targets; however, this may be biased by the possible inclusion of some indirectly regulated genes in our functional target lists for each TF. Indeed, when considering only putative direct target genes for p65 defined by ChIP-seq evidence for promoter binding, this fraction rises to 34%; and by restricting the analysis to only putative direct targets whose expression could also be induced by the p65 TA3 region alone, the fraction of Zbtb7a-regulated promoters using the same cutoff rises further to 43%.

We used microarray analysis of Zbtb7a-knockdown cells to interrogate Zbtb7a-dependent changes in gene expression. Similarly to the regulation of accessibility (Fig 3E and 3F), we found that expression levels of individual genes with Zbtb7a-bound promoters could be both reduced but also increased by knockdown of Zbtb7a (S4A and S4B Fig). Zbtb7a-dependent increased expression is generally associated with increased promoter accessibility (S4C Fig), whereas the converse is true for genes that are repressed by Zbtb7a. Promoters that exhibit Zbtb7a-dependent repression in fibroblasts are moderately enriched for a small set of sequence motifs, possibly reflecting TFs that are amenable to, or participate in, repression by Zbtb7a (S4D Fig). To corroborate this, we reanalysed several publicly available gene expression datasets describing the effect of Zbtb7a deficiency: consistent with our findings, in most cases, the proportions of Zbtb7a-activated and -repressed genes were similar (S4E Fig). At Zbtb7a-dependent promoters, we found that the magnitude of Zbtb7a-regulated accessibility correlates strongly with the magnitude of Zbtb7a-dependent gene expression (S4F Fig), consistent with the notion that regulation of promoter accessibility represents a mechanistic step in gene activation.

Using the microarray data, we examined the effect of Zbtb7a depletion on the expression of the experimentally defined target genes of the Zbtb7a-utilising TFs identified above. In line with their impaired promoter accessibility, we found that the expression levels of functional TF target genes were detectably reduced in Zbtb7a-knockdown cells. Furthermore, although the magnitude of this effect was moderate for many TF target genes, it was most pronounced at the subset of target genes for each TF that displayed Zbtb7a-regulated promoter accessibility (Fig 4D).

To independently confirm the link between gene activation by cJun, Tead2, Runx2, Cebpd, and NFκB and regulation by Zbtb7a, we validated a set of Zbtb7a-dependent target genes for each TF using Zbtb7a-knockout fibroblasts. To avoid indirect effects that can arise from differently derived cell lines, we acutely restored Zbtb7a by stable transduction into the Zbtb7a-knockout cells. For all Zbtb7a-utilising TFs, selected target genes exhibited reduced expression in Zbtb7a-knockout cells compared to congenic controls, and their expression was partially or completely rescued by experimental restoration of Zbtb7a (S5 Fig).

The set of Zbtb7a-utilising TFs described here is undoubtedly incomplete because we pursued only the top-ranking motifs associated with Zbtb7a-bound regions, in a single cell type and under steady-state conditions. Nevertheless, our results demonstrate that Zbtb7a is necessary to regulate promoter accessibility and enable optimal gene expression at the target genes of a diverse but specific set of TFs.

### 5. Zbtb7a transduces regulation of accessibility upstream and independently of transcriptional activation

A straightforward interpretation is that Zbtb7a is required to transduce activation signals from each TF to downstream effector complexes that mediate changes in chromatin accessibility. However, an alternative hypothesis could be that the observed Zbtb7a-dependent changes in accessibility may instead arise as a downstream consequence of transcriptional activation. To address these possibilities, we used p65 TA3 as a model whereby Zbtb7a-dependent regulation of accessibility can be separated from direct transcriptional activation.

**Fig 5:**
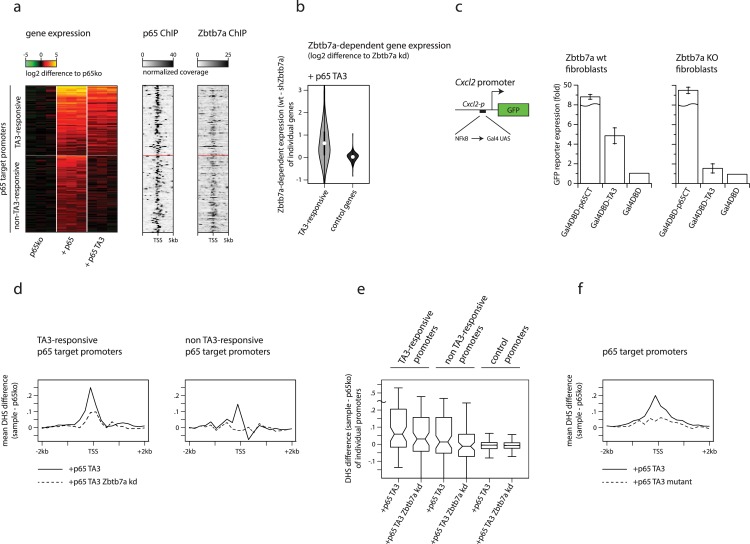
Zbtb7a transduces regulation of accessibility upstream and independently of transcriptional activation. (A) Left: mRNA expression levels of 153 direct p65 target genes (73 “TA3-responsive” plus 80 “non-TA3-responsive”), in TNF-α-treated p65-knockout fibroblasts and in fibroblasts reconstituted with p65 or p65 TA3. Middle: p65 ChIP signals, and right: Zbtb7a ChIP signals, at promoters of p65 target genes in TNF-α-treated normal fibroblasts. mRNA levels are log2 microarray signal differences to non-reconstituted p65-knockout fibroblasts, for 3 biological replicates. (B) mRNA expression differences between TNF-α-treated control and Zbtb7a-knockdown fibroblasts expressing p65 TA3, at distinct gene sets. Dots in violins indicate mean values. Significance of expression difference between TA3-responsive and control genes: *P =* 3.0 × 10^−14^. (C) GFP reporter expression in TNF-α-treated control (left) or Zbtb7a-knockout (right) fibroblasts, expressing p65 C-terminal regions fused to the DBD of Gal4 and cotransfected with plasmids carrying 1 kb promoter sequences from the *Cxcl2* gene, in which NFκB binding motifs are replaced by the Gal4-UAS. Zbtb7a-knockout and congenic-control fibroblasts are both derived on a p53-knockout background, to prevent premature senescence [20]. Error bars indicate SEM. (D) Mean TA3-induced DNase hypersensitivity levels across TA3-responsive (left) or non-TA3-responsive (right) p65 target promoters, in TNF-α-treated control and Zbtb7a-knockdown fibroblasts. (E) DNase hypersensitivity levels induced by p65 TA3 at TA3-responsive, non-TA3-responsive, or control (non-NFκB target) promoters, in TNF-α-treated control and Zbtb7a-knockdown fibroblasts. Induced levels represent differences in mean cut frequencies within ±600 bp surrounding the TSS, compared to p65-knockout fibroblasts. Lines in boxplots indicate median values; whiskers extend to the most extreme data within 1.5× the IQR from the box; outliers are not shown. Significance of difference to Zbtb7a knockdown at non-TA3-responsive *P =* 4.7 × 10^−2^. (F) Mean induced DNase hypersensitivity levels across p65 target promoters, in TNF-α-treated fibroblasts expressing p65 TA3 or a loss-of-function TA3 mutant that does not interact with Zbtb7a (“p65 TA3 mutant”). Additional details of statistical analysis are provided as Supporting information, and numerical values underlying figures are reported in S1 Data. ChIP, chromatin immunoprecipitation; DBD, DNA-binding domain; GFP, green fluorescent protein; IQR, interquartile range; NFκB, nuclear factor kappa B; SEM, standard error of the mean; TNF-α, tumour necrosis factor alpha; TSS, transcription start site.

We set up a cellular system in which normal levels of p65 expression can be stably restored into p65-knockout fibroblasts by retroviral transduction and cell sorting (S6A Fig); we then used this system to establish fibroblast cell lines expressing similar levels of normal and engineered variants of p65 (S6B Fig), which can be directly compared to each other and to the parental p65-deficient cells. Using this approach, we performed microarray analysis to examine gene activation by the p65 TA3 variant lacking direct activation domains (Fig 1A). Reconstitution with p65 TA3 is sufficient to restore activation of around half of all p65 direct target genes in TNF-α-stimulated p65-knockout fibroblasts (Fig 5A). For the majority of these TA3-responsive genes, restoration of gene expression is impaired upon simultaneous knockdown of Zbtb7a (Fig 5B), confirming the general requirement for Zbtb7a for context-dependent activation by p65 TA3. As an independent test, and to formally exclude any indirect effects of Zbtb7a knockdown on upstream NFκB pathway activation, we also assayed the ability of an artificially tethered TA3 domain to induce activation of a *Cxcl2* promoter–reporter vector in Zbtb7a-knockout cells. We fused the C-terminus (CT) or the TA3 region from p65 to the DNA-binding domain from yeast Gal4 and replaced the NFκB binding motifs in the *Cxcl2* promoter with a Gal4 binding sequence (Gal4-UAS). Similarly to the activities of p65 variants on the intact *Cxcl2* promoter (Fig 1E), both Gal4-p65CT and Gal4-TA3 were able to induce activation of the *Cxcl2* promoter containing a Gal4-UAS site in control fibroblasts, and activation of the plasmid reporter by p65CT (containing the direct activation domains TA1 and TA2) was not detectably impaired by the absence of Zbtb7a. In contrast, however, the activity of Gal4-TA3 was completely abolished in Zbtb7a-knockout cells (Fig 5C).

To separate transcriptional activation from regulation of promoter accessibility, we focused on those p65 target promoters for which the activity of p65 TA3 is insufficient to induce transcription. We observed that, at many of these “non-TA3-responsive” promoters, p65 TA3 is nonetheless able to induce measurable changes in accessibility, in a Zbtb7a-dependent fashion (Fig 5D and 5E). Therefore, these examples establish that Zbtb7a can transduce changes in target site accessibility upstream and independently of transcriptional activation. This conclusion is also supported by the ability of Zbtb7a to regulate accessibility at many enhancer regions (Fig 3F), at which transcription rates are many-fold lower than at promoters [25].

To directly address whether the interaction between a TF activation domain and Zbtb7a drives the regulation of promoter accessibility, we also reconstituted p65-knockout fibroblasts with a form of p65 TA3 bearing the function-impairing mutations that disrupt its interaction with Zbtb7a (Fig 1F, S1E Fig). In these cells, promoter accessibility induced by the TA3 mutant was significantly reduced compared with the level induced by unmutated p65 TA3 (Fig 5F). Lower levels of accessibility can still be observed at some promoters in this situation (S6C, S6D and S6E Fig); however, because the mutations in the TA3 domain reduce but do not abolish binding to Zbtb7a—reduced by approximately 5 to 7 times (based on SILAC ratio; Fig 1H) or by approximately 2 times (based on overexpression and WB; Fig 1I)—it is likely that these residual effects are driven by a remaining weak interaction with Zbtb7a. Nevertheless, these results strongly support a model whereby it is the interaction between TFs (exemplified here by p65 TA3) and Zbtb7a that triggers changes in accessibility (most likely through activation or recruitment of remodeling enzymes; see Discussion).

The strong preference of Zbtb7a for binding to promoters and enhancers (Fig 2A, 2B, 2C and 2D)—and its substantial overlap with NFκB target promoters (Fig 2D, S2A, S2B and S2C Fig) and with target sites for other TFs (Fig 4A)—strongly suggests that interaction with TFs and regulation of accessibility is performed by promoter-bound (or enhancer-bound) Zbtb7a. To test this, we disrupted the consensus Zbtb7a binding motif present in the TA3-responsive *Saa3* promoter and assayed its activity in a reporter plasmid. Deletion of the putative Zbtb7a binding site resulted in significantly impaired reporter activation by p65 TA3 (S6F Fig). In a reciprocal experiment, we generated a reporter vector bearing the approximately 1 kb promoter region of the TA3-responsive *Gem* gene but excluding the consensus Zbtb7a motif, which is present in the first intron. In this case, reporter expression is driven by p65 TA3 only when Zbtb7a binding is experimentally restored (by recruitment to the reporter plasmid as a fusion to the Gal4 DNA-binding domain; S6G Fig). Together, these results support the hypothesis that binding to its target loci is required for Zbtb7a to interact with TFs and regulate promoter accessibility and activation.

### 6. Prebinding of Zbtb7a can specify the responsiveness of promoters and enhancers to induction of accessibility by TFs

What is the role of Zbtb7a at promoters and enhancers that are nonregulated or inactive under steady-state conditions (Figs 2F, 3C, 3E and 3F)? We hypothesised that these may represent sites that are utilised in response to distinct signals, or in different developmental contexts, but that are unbound by the appropriate effector TF(s) in fibroblasts. In this case, the presence of Zbtb7a could serve to impart responsiveness to these elements, in readiness for stimulus- or differentiation-induced TF recruitment.

**Fig 6:**
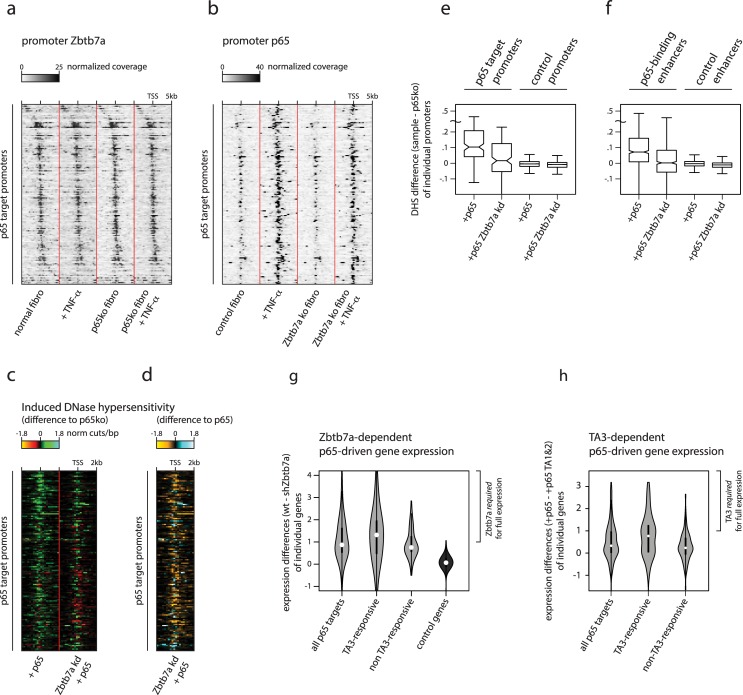
Prebinding of Zbtb7a can specify the responsiveness of promoters and enhancers to induction of accessibility by TFs. (A) Zbtb7a ChIP signals at p65 target promoters in untreated (left) and TNF-α-treated (left centre) normal fibroblasts, and untreated (right centre) and TNF-α-treated (right) p65-knockout fibroblasts. Promoters are sorted in order of Zbtb7a peak position. (B) p65 ChIP signals at p65 target promoters in untreated (left) and TNF-α-treated (left centre) control (congenic p53-knockout) fibroblasts, and untreated (right centre) and TNF-α-treated (right) fibroblasts derived from Zbtb7a-knockout fibroblasts. Promoters are sorted in order of Zbtb7a peak position. (C, D) Induced DNase-I hypersensitivity at p65 target promoters, in TNF-α-treated control or Zbtb7a-knockdown fibroblasts expressing p65. DNase hypersensitivity levels are shown at individual promoters as the differences to the levels observed in non-reconstituted p65-knockout fibroblasts (C), or as the differences between Zbtb7a-knockdown and control fibroblasts (D). (E, F) DNase-I hypersensitivity levels induced by p65, at p65 target and control promoters (E) or enhancers (F), in TNF-α-treated control and Zbtb7a-knockdown fibroblasts. Induced levels represent differences in mean cut frequencies within ±600 bp surrounding the TSS for promoters, or surrounding enhancer midpoints, compared to p65-knockout fibroblasts. Lines in boxplots indicate median values; whiskers extend to the most extreme data within 1.5× the IQR from the box; outliers are not shown. Significance of difference to Zbtb7a knockdown: p65 target promoters *P =* 2.4 × 10^−8^; p65-binding enhancers *P =* 1.1 × 10^−134^. (G) mRNA expression differences between TNF-α-treated control and Zbtb7a-knockdown fibroblasts expressing p65, at distinct gene sets. Genes whose expression levels are most reduced by Zbtb7a knockdown are indicated as those for which Zbtb7a is required for full expression. Dots in violins indicate mean values. Significance of expression difference between TA3-responsive and control genes: *P =* 1.6 × 10^−26^. (H) mRNA expression differences between TNF-α-treated fibroblasts expressing p65 and those expressing the p65 TA1&2 variant, at distinct gene sets. Genes whose expression levels are most reduced in p65 TA1&2-expressing cells (lacking the TA3 region) are indicated as those for which TA3 is required for full expression. Dots in violins indicate mean values. Significance of expression difference between TA3-responsive and control genes: *P =* 2.3 × 10^−12^. Additional details of statistical analysis are provided as Supporting information, and numerical values underlying figures are reported in S1 Data. ChIP, chromatin immunoprecipitation; IQR, interquartile range; TF, transcription factor; TNF-α, tumour necrosis factor alpha; TSS, transcription start site.

To test this hypothesis, we again focused on target genes of NFκB p65 as a model. In p65-knockout fibroblasts, NFκB target genes are nonexpressed and require restoration of p65 as well as cellular stimulation to be activated. We therefore examined whether Zbtb7a is already prebound at p65 target promoters in this cellular setting. We performed ChIP-seq for Zbtb7a in p65-knockout and normal fibroblasts, both in resting conditions and after stimulation of NFκB pathway activation using TNF-α. Zbtb7a binding to inactive p65 target promoters was completely undiminished in p65-knockout cells as well as in unstimulated fibroblasts (Fig 6A). Moreover, whereas TNF-α stimulation strongly induced recruitment of p65 to its target sites in promoters and enhancers (Fig 6B), the levels of bound Zbtb7a at the same regions remained unchanged. Therefore, Zbtb7a prebinds to the target promoters and enhancers of its “client” TF p65, independently of p65 binding.

At p65 target promoters, which are normally loaded with prebound Zbtb7a, binding of p65 triggers changes in promoter accessibility and gene activation. We therefore examined whether the presence of Zbtb7a is required for, or facilitates [16], inducible p65 recruitment. We performed ChIP-seq for p65 in Zbtb7a-knockout fibroblasts, and in congenic-control cells, during TNF-α stimulation. The complete absence of Zbtb7a had no detectable effect on stimulus-driven p65 recruitment to >93% of its normal target sites (Fig 6B), ruling out its requirement for NFκB pathway activation and indicating that client TF binding can occur independently of the presence of Zbtb7a.

Despite normal recruitment of p65 to its target regions in the absence of Zbtb7a, p65 is impaired in its ability to trigger changes in accessibility upon Zbtb7a knockdown. In p65-knockout cells, the basal level of accessibility at inactive NFκB target promoters is very low (S7A Fig), but this is induced by restoration of p65 and TNF-α stimulation. Similar to our observations using the isolated TA3 region, simultaneous knockdown of Zbtb7a was able to strongly reduce the ability of reintroduced p65 to trigger increased accessibility at its target promoters and enhancers in p65-knockout cells (Fig 6C, 6D, 6E and 6F, S7 Fig). Therefore, the presence of prebound Zbtb7a at future TF target sites is required to allow newly recruited client TFs to trigger accessibility changes.

Finally, we examined the scope and influence of Zbtb7a-dependent regulation on normal p65-driven gene expression. Like most other TFs, p65 contains autonomously acting, direct transcription activation domains (TA1 and TA2), so it was pertinent to assess the extent to which indirect, context-dependent activation transduced by Zbtb7a can additionally contribute to normal TF-driven gene expression levels. As already shown above (Fig 4D, S1O Fig), overall levels of p65 target gene activation are reduced upon knockdown of Zbtb7a. Furthermore, by dividing p65 target genes according to their activation by p65 TA3 alone (Fig 5A), we found that the majority of TA3-responsive genes are strongly dependent on Zbtb7a, even when assaying the activity of full-length p65 (Fig 6G). In a reciprocal experiment, we reconstituted p65-knockout fibroblasts with a p65 variant containing only TA1 and TA2—and lacking all TA3 regions—and compared its ability to activate p65 target genes to that of intact p65. This form of p65 is able to strongly activate expression from reporter plasmids (Fig 1B and 1E; see also [9,11]). Nevertheless, more than half of TA3-responsive genes displayed significantly reduced expression when driven by the p65 TA1&2 variant (Fig 6H). In summary, TA3- and Zbtb7a-transduced regulation plays an important contribution to p65-driven activation of a subset of its target genes, which is not accomplished by its direct activation domains alone. It seems likely that many of the other TFs that utilise Zbtb7a may have a similar pattern of behaviour.

## Discussion

**Fig 7:**
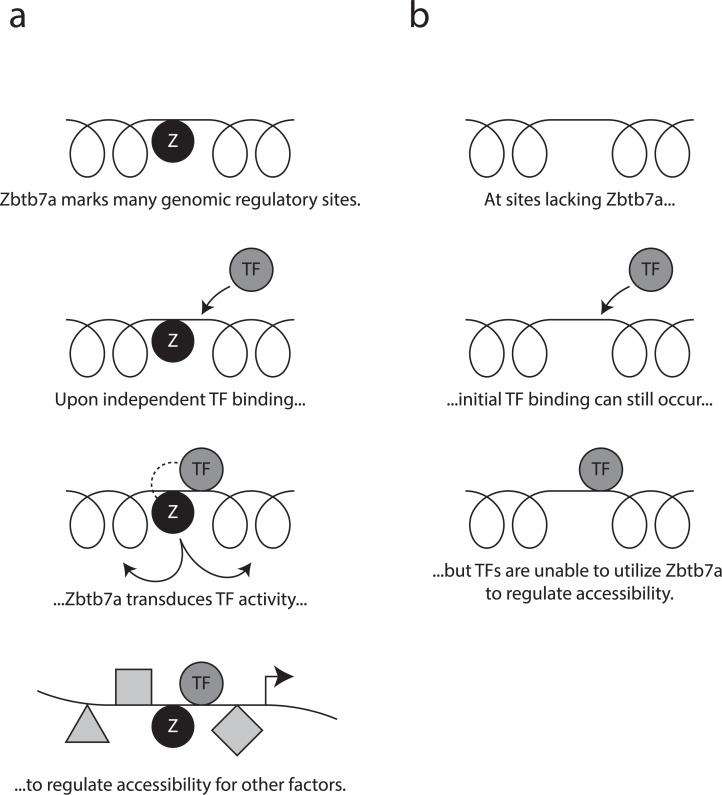
Scheme of Zbtb7a function at gene-regulatory elements. Cartoon outlining role of Zbtb7a described in this paper. Principal experimental evidence for each depicted step is shown in the indicated figures. (A) Top row: Zbtb7a binds to many genomic regulatory sites, including promoters and enhancers (see Fig 2C and 2D). Zbtb7a binding is independent of the presence of client TFs, and binding may occur before client TF recruitment (see Fig 6A). Second row: Zbtb7a-utilising client TFs bind independently to neighbouring genomic sites. This may occur under normal, steady-state conditions or in response to stimulation (see Figs 4A and 6B). Third row: Zbtb7a transduces TF-dependent changes in local chromatin accessibility (see Figs 4C, 5D, 6C, 6D, 6E and 6F). In the case of p65, this is triggered by the interaction between Zbtb7a and the TA3 region of p65 (see Fig 5F). Bottom row: Zbtb7a-transduced accessibility allows binding of secondary TFs and contributes to normal gene activation (see Figs 4D, 6G and S3L and S3M Fig). (B) At genomic sites that lack Zbtb7a binding (which could occur due to the natural genomic distribution of Zbtb7 or through experimental manipulation), client TF binding is unimpaired (see Fig 6B), but Zbtb7a-dependent regulation of accessibility is abolished (see Figs 4C, 6C, 6D, 6E and 6F). TF, transcription factor.

Together, our data show that Zbtb7a associates with a large set of genomic promoters and enhancers, where it acts as a prebound transducer, which is required for many TFs to induce changes in accessibility (summarised in Fig 7). Using NFκB p65 as a model Zbtb7a-utilising TF, we find that binding of Zbtb7a and of its “client” TF are independent events and that induction of accessibility is triggered by the interaction between a specific TF domain and Zbtb7a. Moreover, at the target genes for a diverse set of TFs, involved in multiple biological processes, Zbtb7a is required for regulation of promoter accessibility and for normal gene expression.

Numerous TFs—including “pioneer” factors—have been shown to be able to induce changes in accessibility at their target sites, but in most cases, it is unclear how this effect is triggered [6–8]. Our results indicate that many TFs may not themselves carry this functionality nor independently recruit and activate the remodeling enzymes required; instead, they rely on the presence of Zbtb7a as a prebound “transducer” at their target sites. This notion implies that the set of target sites amenable to regulation upon binding by a particular TF may be predetermined in a given cell type by the genomic distribution of Zbtb7a, or other “transducer” factors: this could act to control the scope of activity of TFs in a given cell type and might even represent a limitation to the possible targets of pioneer TFs that function using the same (or related) mechanisms.

Zbtb7a has previously been characterised to be involved in both repression and activation of several individual model promoters, with its described effect(s) varying according to the system studied [17]. Our global analysis of Zbtb7a binding and function is in agreement with this: we find that similar proportions of Zbtb7a-regulated promoters appear to be repressed or activated in a Zbtb7a-dependent fashion, and regulation is generally direct in both situations (based on binding of Zbtb7a and enrichment for Zbtb7a sequence motifs). We obtained the same results using independent, publicly available data (S4E Fig). However, our data indicate that Zbtb7a does not behave as a direct transcriptional activator (in line with its lack of any described activation domain)—because its presence at many nonexpressed gene promoters is not alone sufficient to drive gene expression—and that it instead functions by transducing TF-driven changes to promoter accessibility. It remains to be determined whether gene repression by Zbtb7a is accomplished using a similar mechanism. Regulation of accessibility could be envisaged to enable either activation or repression at a given promoter, depending on whether the outcome increases or decreases the availability of specific TF motifs, and according to the downstream recruitment of activator or repressor proteins. Nevertheless, at NFκB target promoters in fibroblasts, Zbtb7a-dependent changes in accessibility are overwhelmingly associated with gene activation (Figs 3G, 5B and 6G), hinting that these promoters may be preconfigured to respond in a consistent fashion. Likewise, the other TFs that we investigated also appear to utilise Zbtb7a predominantly to mediate gene activation, and a similar situation may also apply at genes activated by the TF GATA1 in erythroid cells in which Zbtb7a was previously described to preferentially occupy the promoters of GATA1-activated genes [26] (although a role in repression of other GATA-1 targets has also since been reported [27]).

The structural events that enable Zbtb7a to trigger changes in promoter accessibility remain to be determined. The POZ/BTB domain, present at the N-terminus of Zbtb7a, is a highly conserved interaction and dimerisation domain found in many metazoan DNA-binding proteins. Shortly after its original description [28,29], it was conjectured that many nuclear POZ domain proteins may represent “regulators of chromatin folding rather than direct transcriptional regulators” [30]. This notion fits well with our proposed mode-of-action of Zbtb7a. However, the molecular effectors of Zbtb7a, and indeed of many POZ-ZF proteins, are not well characterised. Most structural insights into POZ function so far have come from studies of the mammalian TF B cell lymphoma 6 (Bcl6), which binds either to NCoR/SMRT or BCoR cofactors through an interaction groove located within the dimerisation interface of its POZ domain [31,32]. Notably, though, the Bcl6-binding domains of SMRT and of BCoR do not share any significant sequence similarity to each other, and moreover, the sequence of the interacting groove within the Bcl6 POZ domain is not conserved in other POZ-ZF proteins [33]. Thus, although the POZ domain may represent a common cofactor-interaction module for POZ-ZF proteins, its binding targets can be highly protein specific, and a single POZ domain can interact with diverse cofactors. The POZ domain of Zbtb7a itself has been reported to interact with several proteins that can act as subunits of nucleosome remodeling complexes [27]. These could provide a direct, mechanistic link to the regulation of promoter accessibility, although it remains to be resolved whether they represent interactions with a single complex or with subunits of multiple, distinct complexes. Prominent among reported interactors are subunits of NuRD (nucleosome remodeling and deacetylase) complexes, which have been best characterised for their role in gene repression [34] (although some reports have found that they can associate with promoters or enhancers of active genes or even function as activators [35,36])—therefore, it remains to be established whether remodeling by variants of these complexes could also be responsible for Zbtb7a-mediated promoter activation.

A key aspect of the “transducer” model of Zbtb7a function is our finding that its gene-regulatory activity is only triggered upon interaction with other TFs, exemplified by the TA3 region of p65. How could such an interaction enable Zbtb7a activity? One plausible possibility is suggested by the known ability the POZ and ZF domains of Zbtb7a to mutually interact [37]. Our in vitro interaction experiments suggest that Zbtb7a contacts the TA3 region through its C-terminal ZF domain (similarly to other POZ-ZF proteins that have been described to interact with collaborating TFs [38–40]), implying that binding of TA3 (or other TF domains) to the Zbtb7a ZF domain may displace the prebound POZ domain, thereby allowing it to bind and recruit other effector molecules. An analogous displacement of prebound p300 has been reported upon binding of the TF Myc to the ZF domain of another POZ-ZF protein, Miz [38]. This model, or related alternatives based on competitive binding to the same domain of Zbtb7a, may also explain how mutations that reduce the strength of binding by TA3 (by approximately 2 to 7 times; Fig 1H and 1I) could result in an all-or-nothing functional outcome. Future studies will be needed to establish whether this or another mechanism underlies the transducer function of Zbtb7a.

Lastly, Zbtb7a has been strongly linked to cancer development and progression. Intriguingly, though, Zbtb7a has been found to behave both as an oncogene and as a tumour suppressor, depending on context [17]. Based on our results, it seems likely that the widespread role of Zbtb7a as a potential cofactor for multiple TFs in diverse pathways may contribute to the pleiotropic effects exhibited by cells with disrupted or misregulated Zbtb7a.

## Materials and methods

### Cell culture

Mouse 3T3 fibroblasts used in these experiments were derived from wild-type, p65-knockout, p53-knockout, and p53-/Zbtb7a-double-knockout mice and were treated with 5 ngml^−1^ recombinant mouse TNF-α to stimulate NFκB pathway activation (1 hour before analysis, unless otherwise specified). For reporter assays, fibroblasts were transiently transfected by electroporation or using lipofectamine, and HEK-293 cells were transfected using CaPO_4_. Stably transduced fibroblast cell lines were generated with retroviral vectors using supernatants from transfected Ecotropic-Phoenix packaging cells. Reconstituted p65-knockout fibroblasts were infected with serial dilutions of retroviral supernatants and analysed by flow cytometry; samples with comparable expression levels of co-expressed Tomato protein were chosen and sorted for low and homogeneous Tomato levels, and the levels of expressed p65 variant proteins were assayed by intracellular staining using an antibody specific for the DBD of p65, present in all variants; all cell lines used expressed similar levels of p65 variants to the level of p65 in wild-type fibroblasts (see S6B Fig). Zbtb7a expression was experimentally restored in p53-/Zbtb7a-double-knockout fibroblasts by retroviral infection and sorting for low levels of co-expressed Tomato protein (corresponding to around 10- to 20-fold overexpression of Zbtb7a-encoding mRNA) (see S5A Fig). All cells were grown in DMEM supplemented with 10% fetal bovine serum (FBS). For preparation of nuclear extracts for SILAC MS analysis, dialysed (3.5 kDa cutoff) FBS was used, and lysine- and arginine-deficient DMEM was supplemented either with normal (“light”) or isotopically labelled (“heavy”: lysine ^13^C6 plus arginine ^15^N4) amino acids.

### Plasmids

All p65 variants were derived from the coding sequence of mouse p65 and were expressed using pMY-ires-Tomato (a retroviral vector driving co-expression of red fluorescent Tomato protein) or pCDNA3. p65 DBD: amino acids (aa) 1–305; p65 TA3: aa 1–440,475–519; p65 TA1&2: aa 1–305,441–549; p65 TA3 mutant: substitution of aa 361–370 in TA3 to alanine residues. The positions of other truncations, deletions, and mutations within p65 are summarised in S1D Fig. To generate Gal4DBD fusion proteins (used in Fig 5C, S1J, S6F and S6G Figs), the DBD of yeast Gal4 (aa 1–147) was fused to the carboxy-termini of p65 (aa 306–549) or TA3 (aa 306–440,475–519), or to full-length Zbtb7a. For BiFC, proteins fused at either the N- or C-termini using a 14aa glycine/serine linker to fragments of the Venus fluorescent protein (V1: aa1–158, V2: aa159–239) were expressed using pCDNA3. Reporter plasmids containing different promoters were based on pGL3-Gfp. “NFκB motif only” promoter: 3x GGGATTCCCC motifs immediately upstream of an artificial minimal promoter (comprising fused fragments from chicken conalbumin and SV40 promoters); *Cxcl2*, *Saa3*, and *Gem* promoters: approximately 1 kb genomic sequences upstream of the TSS (*Cxcl2*: mm9 chr5:91331907-91332973(+); *Saa3*: mm9 chr7:53970968-53971803(-); *Gem*: mm9 chr4:11630585-11631714(+)). The *Cxcl2* promoter was modified in some experiments (Figs 1E and 5C) by replacing the 2 NFκB binding motifs (chr5:91332828-91332859(+)) with the Gal4 UAS sequence; the *Saa3* promoter was modified (S6F Fig) by disrupting the consensus Zbtb7a binding motif (chr7: 53971038-53971045(+); mutated GGGACCCC to AAGCTTCC [mutations underlined]), as well as a consensus motif within the *Gfp* coding sequence; a 5x tandem repeat of the Gal4 UAS sequence was cloned into the *Gem* promoter reporter plasmid (S6G Fig). *Zbtb7a* was stably knocked down using hairpins directed against either GCACAACTACGACCTGAAGAA or GAAGCCCTACGAGTGTAACAT (within the *Zbtb7a* CDS), cloned into the retroviral vector pSirΔ-U6CPuro (which drives hairpin expression from the mouse U6 promoter and confers resistance to puromycin). For control experiments, a hairpin directed against GGCACAAGCTGGAGTACAACT (derived from the *Gfp* CDS) was used. The full-length Zbtb7a coding sequence was expressed in fibroblasts using pMY-ires-Tomato. The Zbtb7a ZNF domain (aa 343–569) was expressed in vitro from pCDNA3. For production of GST fusion proteins, bait protein coding sequences (full p65 CT, p65 TA3, and p65 TA3 mutant) were cloned downstream of the GST coding sequence in pGEX-4T-1.

### Antibodies

Polyclonal antibodies against p65 (c20; sc-372), Cebpb (c19; sc-150), JunD (329; sc-74), and the HA epitope (Y-11, sc-805) as well as monoclonal antibodies against p65 (clone F6; sc-8008) and Zbtb7a (clone 13E9, sc-33683) were from Santa Cruz biotechnology.

### Zbtb7a knockdown

p65-knockout 3T3s were infected using retroviral knockdown vectors targeting either of 2 regions within the *Zbtb7a* CDS or a control region (within the *Gfp* CDS) and were selected for stable transduction using puromycin. For both hairpins targeting *Zbtb7a*, *Zbtb7a* mRNA levels were reduced by 50% to 60%, Zbtb7a protein levels were reduced by around 80%, and TNF-α-induced expression of the TA3-responsive p65 target gene *Cxcl2* was reduced more than 10-fold. Zbtb7a mRNA and protein levels, and expression of *Cxcl2*, were unaffected in control cells using the hairpin targeting *Gfp*.

### Gene expression analysis

For analysis of the expression of individual genes, cDNA was prepared from total cellular RNA by reverse transcription using random hexamer primers, and gene expression was assayed using quantitative real-time PCR with gene-specific fluorescent probes. Gene expression was normalised to the expression level of *Tbp*. For microarray analysis, RNA samples were prepared and processed using Qiagen RNeasy purification kits. Sample processing and microarray hybridisation was performed using standard procedures (KFB, Regensburg).

### ChIP and ChIP-seq

For ChIP, fibroblasts were washed in PBS and fixed at room temperature with 2 mM di-succinimidy-glutaraldehyde (DSG) for 45 minutes, washed extensively in PBS, and fixed again with 1% formaldehyde for 15 minutes. Cells were then washed extensively in ice-cold PBS, and nuclei were released by incubation for 5 minutes in ice-cold L1 buffer (50 mM Tris [pH 8], 2 mM EDTA, 0.1% NP40, 10% glycerol) followed by 5 minutes of centrifugation at 1,000 g. Nuclei were lysed in L2 buffer (50 mM Tris [pH 8], 5 mM EDTA, 1% SDS) at a concentration of 5 × 10^7^/ml. Chromatin in the supernatant was fragmented to a size range of approximately 300 to 700 bp using a tip sonicator, and insoluble debris was removed by centrifugation. Chromatin was diluted 10-fold in DB (50 mM Tris [pH 8], 5 mM EDTA, 200 mM NaCl, 0.5% NP40) and precleared with 27 μl/ml protein-A sepharose (for ChIP using rabbit IgG) or protein-G sepharose (for mouse and hamster IgG) for at least 1 hour at 4°C. Precleared chromatin was incubated with antibodies at a concentration of 2μg/ml overnight at 4°C, immunoprecipitated for 30 minutes using 10 μl/ml protein-A or -G sepharose, and sequentially washed 6 times with ice-cold NaCl wash buffer (20 mM Tris [pH 8], 2 mM EDTA, 500 mM NaCl, 1% NP40, 0.1% SDS) followed by 3 times with ice-cold TE (50 mM Tris [pH 8], 2 mM EDTA). Immunoprecipitated chromatin was released by incubation at room temperature in buffer EB (50 mM Tris [pH 8], 2 mM EDTA, 2% SDS), and cross-links were reversed by overnight incubation at 65°c. ChIP and input DNA were both purified using Qiagen MinElute PCR purification kits. Recovery at individual promoters was determined using quantitative real-time PCR with amplicon-specific fluorescent probes and was normalised by parallel measurement of input DNA samples. For ChIP-seq, DNA was refragmented as required to achieve a mean size of 300 bp using a water-bath sonicator. Sequencing library preparation was performed using standard procedures (genecore unit, EMBL), and samples were sequenced using single-end reads.

### DHS sequencing

For mapping of DNase hypersensitive sites, fibroblasts were washed twice in ice-cold PBS, pelleted by centrifugation for 5 minutes at 500 g, and resuspended in ice-cold buffer A (15 mM Tris [pH 8], 15 mM NaCl, 60 mM KCl, 1 mM EDTA, 0.5 mM EGTA, 0.5 mM spermidine, 0.3 mM spermine, 2 mM DTT) at a concentration of 5 × 10^6^/ml. Nuclei were released by the addition of an equal volume of buffer A plus 0.2% NP40 and incubation for 8 minutes at 4°C, then washed once in buffer A, and pelleted by centrifugation for 5 minutes at 500 g. Separate pellets of 5 × 10^6^ nuclei were resuspended in 600 μl of 37°C digestion buffer (buffer A plus 6 mM CaCl2 plus 75 mM NaCl) and digested with varying amounts of DNaseI (typically 40, 80, and 160 units of DNaseI per 600 μl, preincubated for 3 minutes at 37°C) for 150 seconds at 37°C. Digestion reactions were stopped by the addition of 700 μl of stop solution (50 mM Tris [pH 8], 100 mM NaCl, 0.1% SDS, 100 mM EDTA, 20 mg/ml RNase A, 0.5 mM spermidine, 0.3 mM spermine) and incubation for 15 minutes at 55°C, followed by addition of 200 μg proteinase K and incubation for an additional 2 hours at 55°C. Digestions were emulsified with an equal volume of phenol/chloroform/IAA, and the aqueous phase containing digested DNA was collected after centrifugation. NaCl was added to a final concentration of 0.8 M, and digested DNA samples were loaded onto a 10% to 40% sucrose gradient and separated by overnight centrifugation at 90,000 g. Fractions were collected and analysed by agarose gel electrophoresis and staining with SYBR green, and fractions containing fragments <600 bp (with an average size of around 200 bp) were pooled and used for sequencing. Sequencing library preparation was performed using standard procedures (genecore unit, EMBL), and samples were sequenced using single-end reads.

### Reporter assays and BiFC

Fibroblasts were transfected with reporter plasmids, and HEK-293 cells were cotransfected with BiFC plasmids; the fluorescence intensity of GFP-expressing cells was quantified by flow cytometry after 24 to 48 hours.

### GST pull-down and MS

GST fusion protein baits were produced in BL21-codon plus *E*. *coli* and purified to approximately 90% purity (based on inspection of coomassie blue-stained gels) using glutathione sepharose. For pull-downs of in vitro expressed proteins, Zbtb7a fragments were expressed using T7-coupled transcription and translation (TnT, Promega) in the presence of ^35^S-methionine. Expressed proteins were incubated with immobilised bait proteins in NETN buffer (100 mM NaCl, 1 mM EDTA, 20 mM Tris, 0.5% NP-40 [pH 8.0] plus protease inhibitors [“complete” Roche]) at 4°C, washed 3 times in ice-cold NaCl wash buffer (250 mM NaCl, 1 mM EDTA, 20 mM Tris, 0.5% NP-40 [pH 8.0]), and bound proteins were directly analysed by SDS-polyacrylamide gel electrophoresis (PAGE). Gels were stained with coomassie brilliant blue R-250, dried, and exposed using a storage phosphor screen for up to 48 hours to detect ^35^S-labelled proteins. The intensity of the coomassie blue signal was used as a loading reference for bait proteins. For pull-downs of cellular proteins, nuclear extracts were prepared from HeLa S3 cells or 3T3 fibroblasts as described [41] and incubated with immobilised bait proteins for 4 hours at 4°C. Bound proteins were washed 4 times with ice-cold wash buffer (20 mM Hepes [pH 7.6], 1 mM EDTA, 10% glycerol, 0.1% NP40, 100 mM KCl) and released by incubation in elution buffer (20 mM Hepes [pH 7.6], 1 mM EDTA, 5% glycerol, 0.1% NP40, 100 mM KCl, 0.2% sarkosyl) for 2 hours at 4°C. Eluted proteins were separated by SDS-PAGE and analysed by MS. For identification of differentially binding proteins by SILAC, nuclear extracts from cells grown using “heavy” and “light” labelled amino acids were processed in parallel using different baits and mixed immediately after elution. Each nuclear extract was split and processed twice in a “label-swap” setup, so that “heavy” and “light” extracts were each used with each of the 2 baits. Four biological replicates were performed for HeLa S3 cells and for 3T3 fibroblasts, and overall SILAC ratios were calculated as the mean ratio of all replicates in which each protein was detected.

### Analysis of genomic datasets

Sequencing reads were aligned to the mouse reference genome (mm9) using bowtie ([42]; with options -v 2 -a -m 5—tryhard), and statistically excess reads mapping to the exact same location (based on the local level of coverage)—representing likely PCR artefacts—were removed. Mapped read coordinates were extended to the mean fragment size of the sample DNA (300 bp for ChIP, 200 bp for DHS recovered DNA, 1 bp for identification of individual DNase cut sites), and the coverage at each genomic interval was calculated as the mean number of overlapping fragments per base pair (allowing fractional contributions from non–uniquely mapping reads) after normalising datasets to a nominal sequencing depth of 20 million reads. Enriched “peaks” were identified in combined datasets using macs1.4 ([43]; with options -p 1e-4—nomodel—shiftsize = 150 [for ChIP] or 100 [for DHS]—keep-dup = all), using input DNA sequencing data as background and with high-confidence peaks defined as those with a predicted −10log_10_(*P* value) greater than 50. Zbtb7a ChIP-seq datasets exhibited consistently higher backgrounds and lower peak heights when compared to p65 ChIP-seq datasets, likely reflecting differences in ChIP efficiency; nevertheless, high-confidence Zbtb7a peaks exhibited strong correlations between biological replicate datasets (derived from independently cultured cells; Pearson’s r > 0.87) and between parallel datasets generated from untreated and TNF-α-treated cells (Pearson’s r > 0.91), and the Zbtb7a binding sequence motif could be identified at 66% of Zbtb7a peaks, arguing that the signal is highly specific. DHS datasets were normalised (so that differences in the fraction of reads at background, non-hypersensitive regions do not affect the measurement of accessibility at DHS sites) by scaling the coverage so that the number of read-starts (corresponding to DNase cut sites) mapping under predicted DHS peaks was equal for each sample. Note that this conservative approach is valid if the mean accessibility of all DHS regions genome wide is assumed to be approximately equal in all samples; it will underestimate DHS changes between samples if the mean accessibility changes consistently at all, or a large fraction of, DHS sites.

Differences in gene expression were calculated using the means of microarray RMA-normalised signals from 3 biological replicates per experimental group, and genes defined as differentially expressed were required to be significant at a threshold of *P <* 0.05 using unpaired, two-tailed Student *t* tests between groups (based on the described normal distribution of microarray replicate measurements [44]). Subsets of promoters were defined according to criteria based on both microarray expression data and ChIP-seq data: TA3-responsive promoters were defined as those with affymetrix signal difference for (“+p65 TA3”—“p65ko”) ≥0.5 and (“+p65”—“p65ko”) ≥0.4, and with a high-confidence p65 peak within 2 kb. TA3-nonresponsive promoters were those with affymetrix signal difference for (“+p65”—“p65ko”) ≥0.5 and (“+p65 TA3”—“p65ko”) <0.4, and a high-confidence p65 peak within 2 kb. p65 direct target promoters were the union of TA3-responsive promoters and TA3-nonresponsive promoters.

Known and de novo enriched TF motifs at ChIP-seq peaks and promoter subsets were identified using Homer [45]. To analyse DNase footprints at TF motifs, motif instances were first identified within changing DHS regions using Homer, and the patterns of observed DNase cut sites at each nucleotide position surrounding each motif were divided by the mean cut frequencies at all instances of the same surrounding 6 bp sequence across all DHS regions (to avoid any influence of DNase cleavage preferences [22,46]). Note that cut frequencies can be both increased as well as reduced within the TF motif (after correction for cutting biases), likely depending on the exposure of individual nucleotides induced by TF binding, as described [21,47].

To determine the overlap of ChIP-seq data with particular genomic features (Fig 2C), promoters and transcript end sites were defined as the genomic intervals within 1 kb of RefSeq annotated transcript starts and ends, and enhancers were specified as the genomic intervals within 1 kb of the summits of fibroblast H3K4me1 ChIP-seq peaks; the remaining genomic regions were assigned as either exons, introns, or intergenic according to RefSeq annotations, and the fraction of the summits of ChIP-seq peaks that fall within each defined region were calculated.

Promoters with Zbtb7a-regulated promoter accessibility (Fig 3E, 3F and 3G, S3G Fig) were defined as those exhibiting greater changes in DNase hypersensitivity in the 1 kb interval upstream of each TSS than the changes exhibited by 95% or 99% of Zbtb7a-negative promoters, when comparing DHS datasets from normal and Zbtb7a-knockdown fibroblasts. Promoters with Zbtb7a-regulated gene expression (S4A and S4B Fig) were defined as those with an affymetrix signal difference for (“+p65 TA3”—“shZbtb7a + p65 TA3”) ≥0.5 (indicated as “+++” in Fig 6B), or (“+p65 TA3”—“shZbtb7a + p65 TA3”) ≥0.2 (indicated as “+” in Fig 6B), that were also significant at a threshold of *P <* 0.05 using unpaired, two-tailed Student *t* tests between groups.

To identify genes whose expression is controlled by TFs belonging to the AP1, Tead, Runx, Cepb, and NF1 families (Fig 4B, 4C and 4D), we retrieved publicly available gene expression datasets from mouse fibroblasts with experimentally manipulated expression of these TFs (consisting of c-Jun-knockout fibroblasts; fibroblasts with induced overexpression of Cebp-α, -β, -δ, or -ε; fibroblasts expressing a constitutively activating form of Tead2; fibroblasts with ectopic expression of constitutively active Runx1, Runx2, or Runx3; and Nf1c-knockout fibroblasts). High-confidence target genes for each TF were defined as the 200 genes exhibiting the greatest TF-dependent change in expression, which were significant at a threshold of *P <* 0.05 using unpaired, two-tailed Student *t* tests between groups. Where more than 1 member each TF family exhibited significant enrichment for Zbtb7a-regulated accessibility, the family member exhibiting the greatest fraction of high-confidence target promoters with Zbtb7a-regulated accessibility is shown in Fig 4C and 4D. Note that Nf1c target promoters did not display any enrichment for Zbtb7a-dependent regulation (suggesting that other Nf1 family member[s] or other TF[s] recognising a similar motif may account for the motif enrichment at Zbtb7a-bound regions in Fig 4A) and are therefore not shown in Fig 4C and 4D. Individual TF target genes that exhibited reduced expression in Zbtb7a-knockdown fibroblasts were independently validated by quantitative PCR in p53-/Zbtb7a-double-knockout fibroblasts, with and without restoration of Zbtb7a expression to control for nonspecific variation between independently derived cell lines.

### Data

Numerical values underlying figures are reported in S1 Data, and individual data are reported in S2 Data.

Microarray datasets generated in this study are available from the NCBI gene expression omnibus (https://www.ncbi.nlm.nih.gov/geo/) through GEO series accession number GSE97468, and sequencing data are available from the NCBI sequence read archive (https://www.ncbi.nlm.nih.gov/sra/) through the SRA study accession numbers SRP103286, SRP103318, and SRP103319.

Public datasets used to identify high-confidence targets of AP1, Tead, Runx, Cepb, and Nf1 family TFs (Fig 4B, 4C and 4D) (GSE2188, GSE11732, GSE12498, GSE15871, and GSE26205); to determine absolute mRNA levels in 3T3 fibroblasts (Fig 2F & S2I Fig) (GSE39524 [sample GSM970853]); to determine Zbtb7a-dependent increases and decreases in gene expression in diverse cell types (S4C Fig) (GSE41839, GSE70680, GSE74977, GSE24889, and GSE46473); and to define enhancer positions in 3T3 fibroblasts (Figs 2C and 3F, S2G Fig) (GSE32380).

### Statistical analysis

Differences between experimental groups were analysed using two-tailed tests without assumption of equal distribution or variance; full details, including individual tests used and *P* values, are reported as Supporting information.

## Supporting information

S1 Data Numerical values underlying main and supporting figures. (XLSX)

S2 Data Individual data for main and supporting figures. (XLSX)

S1 Fig (A, B) DNase-I hypersensitivity levels induced by p65 DBD, p65 or by p65 TA3, at TA3-responsive or control (non-NFκB target) promoters, in TNF-α-treated p65-knockout fibroblasts. DNase-I hypersensitivity levels represent the mean normalised cut frequencies in 200 bp windows and are shown as the differences to the levels observed in p65-knockout fibroblasts, at individual promoters (A), or the mean levels across all promoters in each group (B). (C) Genome browser example tracks of DNase-I hypersensitivity surrounding the promoters of the TA3-responsive *Camp* gene (top), or the control non-NFκB target *Hprt* gene (bottom), in TNF-α-treated p65-knockout fibroblasts expressing p65 variants. Grey signal indicates coverage of recovered DNA fragments; black signal indicates the density of individual DNase-I cut sites. Lower tracks indicate predicted p65 binding peaks and RefSeq genes. (D–F) Identification of functional TA3 subregions. Variants of p65 TA3 were used to reconstitute p65-knockout fibroblasts and assayed by their ability to activate mRNA expression of the endogenous, TA3-responsive *Cxcl2* gene after treatment with TNF-α. Numbers indicate the amino acid positions in full-length mouse p65. (D) Truncations and deletions of p65 TA3 indicate that removals of regions overlapping positions 344–389 result in >50% loss of activity. (E) Replacement of contiguous 10 amino acid regions within p65 TA3 with alanine residues indicates that positions 361–370 and 370–379 are each required for TA3 activity. The replacement mutant 361–370 >10A (third bar) was used as a loss-of-function mutant in the remainder of this study. (F) Expression of subregions of p65 TA3 indicate that the region spanning positions 342–390 (sixth bar) is sufficient to confer 43% of TA3 activity (and up to 50% activity in independent experiments; not shown). Inclusion of additional adjacent downstream regions (342–415; seventh bar) is able to further augment TA3 activity. We refer to positions 342–390 of p65 as the “minimal TA3” region. Error bars indicate SEM. (G–I) Conservation and primary structure of the p65 TA3 region. (G) Top: cartoon of mouse p65 indicating approximate amino acid boundaries of DNA-binding domain, TA3 region (including minimal TA3 region, “3”), TA2 domain (“2”), and TA1 domain (“1”). Middle: percentage amino acid identities between mouse and human p65 sequences in each region. Bottom: selected species in which genes encoding orthologous regions can be detected using the tblastn search algorithm; the TA3 region is found in most placental mammals but not in more distantly related species. (H) Multiple alignment of p65 protein sequences from different mammalian species with the mouse minimal TA3 sequence. Only aligning protein sequences detected using the blastp search algorithm are shown. (I) Primary structure of the p65 TA3 region. Red arrows represent a series of 6–8 amino acid degenerate repeats with the indicated consensus sequence VPAPAP(AS), which surround a conserved, 20–amino acid region of unique sequence at position 355–374, represented by a green box. Truncations or deletions that reduce the number of repeats progressively diminish the activity of TA3 (panels D, F), whereas mutations or deletions within the unique region abolish its activity (panels D, E). Note that the replacement mutant 361–370 >10A within the unique region, used as a loss-of-function mutant throughout the study, retains all the surrounding repeats, which may contribute to its residual, reduced interaction with Zbtb7a (Fig 1H and 1I). (J) Activity of mouse TA3 region in mouse and human cells. GFP reporter expression from plasmids carrying the 1 kb promoter sequence from the TA3-responsive *Cxcl2* gene, with the NFκB binding motif replaced with the Gal4-UAS, measured after transfection into mouse fibroblasts (left) or human HEK-293 cells (right) cotransfected with the indicated p65 C-terminal regions fused to the DBD of Gal4. Note that the increase in fold expression of the GFP reporter is affected both by the strength of the mouse *Cxcl2* promoter and by the relative transfection efficiencies in each of the 2 cell types; nevertheless, the mouse TA3 region consistently displays an even greater activity in human HEK-293 cells than mouse fibroblasts, when compared with the activity of the full p65 C-terminus (containing also the TA1 and TA2 domains). Error bars indicate SEM. (K) Unique peptides detected by nonquantitative MS. Table lists unique peptide counts for proteins detected after GST pull-down using GST-p65 (left), GST-TA3 (left centre), GST-TA3 loss-of-function mutant (“GST-TA3mut,” right centre), and GST alone (right). The upper box highlights proteins preferentially detected in pull-downs using the full p65 C-terminus and includes known interactors (such as CBP/p300 and components of the Mediator complex). The central box highlights proteins with increased coverage in pull-downs using TA3 than in those using TA3mut (although note that MS analysis in this case was nonquantitative). The lower box highlights proteins that were consistently detected in pull-downs using TA3mut and/or GST alone and that thus represent likely promiscuously binding contaminants. (L) Validation of the interaction between endogenous p65 and Zbtb7a proteins by co-immunoprecipitation from TNF-α-treated unmodified fibroblasts using anti-p65, followed by immunoblotting to detect Zbtb7a. Arrowhead indicates Zbtb7a; dotted lines indicate where membrane was cut after transfer to separate Zbtb7a from the heavy chain of the immunoprecipitating antibody (hc). (M) Measurement of in vivo Zbtb7a interaction with the isolated TA3 region of p65 using BiFC. BiFC fluorescence values represent the GMFI of cells co-expressing the indicated proteins tagged with V1 (carboxy-terminal) or V2 (amino-terminal) fragments of Venus fluorescent protein, expressed as a percentage of the GMFI of cells expressing Zbtb7a-V1 plus V2-p65. Error bars indicate SEM. (N) Pull-down of in vitro transcribed and translated, ^35^S-methionine-labelled Zbtb7a ZF fragment using purified GST alone or GST-TA3. Left: coomassie blue staining of input and pull-down to indicate amounts and purities of bait proteins; right: autoradiograph to detect pulled-down Zbtb7a ZF. No interaction was detected between the TA3 region and full-length Zbtb7a or the BTB domain of Zbtb7a (which was previously reported to interact with the p65 DNA-binding domain [16]). (O) Expression of endogenous *Cxcl2* mRNA in TNF-α-treated p65-knockout fibroblasts expressing full-length p65, with or without shRNA knockdown of *Zbtb7a*. mRNA levels are expressed relative to the level in unstimulated cells without *Zbtb7a* knockdown. Statistical analysis is provided as Supporting information, and numerical values underlying figures are reported in S1 Data. BiFC, bimolecular fluorescence complementation; CT, carboxy terminal domain; DBD, DNA-binding domain; GMFI, geometric mean fluorescence intensity; HEK-293, human embryonic kidney cells 293; IP, immunoprecipitation; MS, mass spectrometry; NFκB, nuclear factor kappa B; RefSeq, NCBI reference sequence database; SEM, standard error of the mean; ZF, zinc finger.
(PDF)

S2 Fig (A, B) Genome browser example tracks across the same 1 Mb and 25 kb genomic intervals shown in Fig 2 including the *Camp* (A) and *Col4a1/2* (B) loci, indicating the p65 ChIP signal and locations of predicted p65 binding peaks (green). (C–F) Overlaps of Zbtb7a ChIP-seq peaks with p65 target promoters and ChIP-seq peaks. Number of overlaps between Zbtb7a ChIP-seq peaks and p65 target promoters ± 500 bp (C), all p65-associated promoters ± 500 bp (including many promoters without evidence for regulation of gene expression by p65 in fibroblasts, D) and all genome-wide p65 ChIP-seq peaks (E, F). Curves indicate the distribution of overlaps expected by chance (using a binomial model); grey bars (in panel E) indicate empirically determined distribution of overlaps, using 900 randomisations of the 2 peak datasets retaining interpeak distances; line indicates the observed number of overlaps and calculated *P* value. (G) Co-association of p65 and other TFs at promoters ± 500 bp. The ϕ^2^ statistic (identical to Pearson’s r^2^ correlation applied to binary data) is indicated for pairwise comparisons of overlaps at promoters of p65 and each of 108 encode ChIP-seq datasets for TFs and other factors that are available for mouse genome assembly mm9, plus the set of Zbtb7a peaks from this study. The co-association of p65 with Zbtb7a ranks fourth among all 109 datasets analysed. Similar results were obtained using the Jaccard statistic to quantify co-association (Zbtb7a ranks fifth among all 109 datasets). Note, however, that encode datasets are derived from diverse cell types, which may contribute to reduced data overlap. (H, I) Genomic context of Zbtb7a peaks. (H) Fraction of CGIs that are associated with predicted Zbtb7a ChIP-seq peaks. (I) GC content (left) and ratio of observed/expected CG dinucleotides (right) at all promoters ± 250 bp and at Zbtb7a-associated promoters. (J) The Zbtb7a binding motif is found at both CGI- and non-CGI-associated Zbtb7a ChIP-seq peaks. DNA sequence motifs matching the described binding specificity of Zbtb7a—enriched among promoter-overlapping (top) and enhancer-overlapping (bottom) Zbtb7a ChIP-seq peaks—were identified by de novo motif prediction. Promoters and enhancers were divided according to their overlap (left) or nonoverlap (right) with annotated CGIs. De novo motif prediction at Zbtb7a peaks overlapping CGI promoters and enhancers was performed using Zbtb7a-negative CGI promoters and enhancers as a control set to minimise the influence of GC content and CG dinucleotiodes on motif prediction. (K) Number of peaks overlapping promoters for Zbtb7a and other ChIP-seq datasets. Encode mm9 ChIP-seq datasets were manually curated as either TF (for example, Gata1, JunD, Maf, Pax5, Tal1, etc.), transcriptional cofactor (comprising Chd1, Chd2, Co-rest, Gcn5, Nelf, p300, Sin3A, Tbp, Ubf, Usf1, and Usf2), Ctcf, or Pol-II. Violins indicate the distribution of the number of overlaps of predicted peaks in each dataset with promoter regions; the top line indicates overlaps of Zbtb7a ChIP-seq peaks with promoter regions. Dots in violins indicate mean values. (L) Zbtb7a-associated promoters include those of genes that are nonexpressed under steady-state conditions in fibroblasts. Mean Zbtb7a ChIP signals across promoters with overlapping Zbtb7a ChIP-seq peaks that are expressed in fibroblasts at the indicated mRNA levels (upper panels) and at promoters without any overlapping predicted Zbtb7a peak (“Zbtb7a negative promoters,” bottom panel). (M) Enrichment of selected GO annotations among genes whose promoters are associated with Zbtb7a peaks (first column, “Zbtb7a promoter peak”) or with increased or decreased mRNA expression in control fibroblasts, compared to Zbtb7a-knockdown fibroblasts (second and third columns). Dots indicate statistically significant enrichments (*P* < 5 × 10^−6^; corrected q < 0.05). The most significantly enriched annotations among genes with Zbtb7a-associated promoters are shown, of which some are also enriched among genes exhibiting Zbtb7a-regulated expression. Bars indicate level of enrichment compared to all annotated genes. Additional details of statistical analysis are provided as Supporting information, and numerical values underlying figures are reported in S1 Data. ChIP-seq, chromatin immunoprecipitation sequencing; FPKM, RNA-sequencing fragments per kilobase transcript per million reads.
(PDF)

S3 Fig (A) Target sequences for knockdown hairpins within the *Zbtb7a* coding region. (B) Efficiency of stable mRNA knockdown by hairpins targeting *Zbtb7a* in p65-knockout fibroblasts, assayed by expression of GFP coded by an independent mRNA engineered to contain the same *Zbtb7a*-derived target sequences. Fibroblast cell lines with higher levels of knockdown (up to 90%) could be generated, but they exhibited reduced proliferation and could not be efficiently expanded (in agreement with the senescence described in Zbtb7a-knockout fibroblasts [20]). Error bars indicate SEM. (C) Effect of independent Zbtb7a-knockdown hairpins on TNF-α-induced expression of the TA3-responsive p65 target gene *Cxcl2*, in fibroblasts expressing p65 TA3. In parallel control experiments using hairpins directed against *Gfp*, *Cxcl2* expression levels were unchanged (not shown). Based on the consistency of the inhibition of *Cxcl2* mRNA expression mediated by both hairpins, we used hairpin 1 for all subsequent experiments in this study. (D) Endogenous *Zbtb7a* mRNA levels in p65-knockout fibroblasts after stable knockdown of Zbtb7a, assayed by quantitative PCR. Error bars indicate SEM. (E) Zbtb7a protein levels in p65-knockout fibroblasts (left), upon ectopic expression of an epitope-tagged form of Zbtb7a (centre), or after stable knockdown of Zbtb7a (right), assayed by immunoblotting for Zbtb7a (top), or Gapdh as a control (bottom). Quantitation of the immunoblot signal indicates that Zbtb7a protein levels are depleted by around 70% to 80% in knockdown cells. (F–J) Genome browser example tracks of DNase-I hypersensitivity (F–I) across the same genomic loci shown in Fig 3 and (J) surrounding the *Neto2* gene promoter (Zbtb7a-bound and Zbtb7a-dependent reduced accessibility), in control or Zbtb7a-knockdown fibroblasts, and indicating the Zbtb7a (black) and p65 (green) ChIP signals and the locations of predicted Zbtb7a- and p65 binding peaks. (K) Zbtb7a-associated promoters that do or do not exhibit ongoing regulation of accessibility by Zbtb7a in fibroblasts display comparable levels of Zbtb7a. Mean Zbtb7a ChIP signals across promoters with overlapping Zbtb7a ChIP-seq peaks (upper panels) and at promoters without any overlapping predicted Zbtb7a peak (bottom panel). Promoters are divided according to whether or not they exhibit Zbtb7a-driven increased or decreased accessibility, as in Fig 3E. (L) DNase-I footprints within regions exhibiting Zbtb7a-dependent DNase-I hypersensitivity. Mean cut frequencies across known TF binding motifs are the ratio of observed to expected DNase-I cuts, based on the local 6 bp sequence context (to account for DNase-I cleavage specificity). The set of known TF binding motifs analysed here are those that are enriched among the complete set of all fibroblast-active promoters and enhancers, reflecting targets of TFs that are active in fibroblasts. (M) Zbtb7a dependence of DNase-I footprints. Footprint magnitudes (difference to background of mean observed/expected cut frequencies across 10 bp surrounding motif centre) at known TF binding motifs within Zbtb7a-dependent DHS regions. Green: control fibroblasts; red: Zbtb7a-knockdown fibroblasts. Note that this scoring method may underestimate the signals of asymmetric or complex footprints (such as Ets1 or Rbpj). Error bars indicate SEM. (N, O) stimulus-dependent, p65-dependent, and Zbtb7a-dependent recruitment of Cebpb (N) and JunD (O) TFs to NFκB target promoters, measured by ChIP in TNF-α-treated p65-knockout fibroblasts expressing p65, or in p65-knockout fibroblasts, with or without shRNA knockdown of *Zbtb7a*. The *Ip10*+4kb region represents a control unbound location, and the *Ip10* promoter exemplifies a non-TA3-driven, non-Zbtb7a-dependent gene promoter. ChIP signal is expressed as the percent recovery of input DNA. Error bars indicate range of duplicate samples. Statistical analysis is provided as Supporting information, and numerical values underlying figures are reported in supporting S1 Data. ChIP-seq, chromatin immunoprecipitation sequencing; GFP, green fluorescent protein; NFκB, nuclear factor kappa B; PCR, polymerase chain reaction; SEM, standard error of the mean; TF, transcription factor; WB, western blot.
(PDF)

S4 Fig (A) Fractions of promoters of genes with changed mRNA expression in Zbtb7a-knockdown fibroblasts. Left: promoters without any associated Zbtb7a peak (“Zbtb7a-negative”); right: promoters with associated Zbtb7a peaks. Green/red slices indicate promoters with increased/reduced mRNA expression in control fibroblasts compared to Zbtb7a-knockdown fibroblasts, at *P* < 0.05 and with an affymetrix signal difference of ≥0.5 (indicated as “+++” / “−−−”) or ≥0.2 (indicated as “+” / “−”). (B) Zbtb7a-associated promoters that do or do not exhibit ongoing regulation of expression by Zbtb7a in fibroblasts display comparable levels of Zbtb7a. Mean Zbtb7a ChIP signals across promoters with overlapping Zbtb7a ChIP-seq peaks (upper panels) and at promoters without any overlapping predicted Zbtb7a peak (bottom panel). Promoters are divided according to whether or not they exhibit Zbtb7a-driven increased or decreased gene expression, as in panel A. (C) Zbtb7a dependence of DNase-I hypersensitivity levels at Zbtb7a-negative promoters and at Zbtb7a-associated promoters of genes with expression that is not Zbtb7a regulated, or with Zbtb7a-driven increased or reduced expression. Zbtb7a-dependent DHS levels represent differences in mean cut frequencies within ±600 bp surrounding the TSS, compared to Zbtb7a-knockdown fibroblasts. Dots in violins indicate mean values. (D) DNA sequence motifs that are enriched at Zbtb7a-repressed promoters (defined as genes with increased expression in Zbtb7a-knockdown fibroblasts). Motif enrichment analysis was performed separately using all promoters, Zbtb7a-independent promoters, and Zbtb7a-dependent promoters as background sets, and motifs exhibiting consistent enrichment are shown. Known TF families with specificities matching each motif are indicated; additional letters of matching known motifs that are not present in the identified de novo motif logos are indicated in grey. *Note that the 2 most enriched motifs (resembling the specificities of the Nrf1 and Egr TFs) are similar to each other (identical preferred nucleotides at 7 of 9 overlapping positions). (E) Zbtb7a regulates both gene activation and gene repression. Percentages of genes exhibiting differential expression in Zbtb7a-deficient cells, in publicly available datasets derived from diverse human and mouse cell types. Genes with reduced expression in Zbtb7a-deficient cells (“Zbtb7a-dependent gene activation”) are shown in green, and those with increased expression in Zbtb7a-deficient cells (“Zbtb7a-dependent gene repression”) are shown in red. In all cases, differentially expressed genes were scored as those with >2-fold change in the means of all replicates and *P* < 0.05 calculated by Student *t* test; however, the relative proportions of activated and repressed genes were also comparable across a range of other expression criteria. (F) Zbtb7a dependence of DNase-I hypersensitivity levels at promoters of genes with Zbtb7a-independent expression, or with increasing Zbtb7a dependency of expression levels, divided into equal quartiles (Q1 = least Zbtb7a-dependent; Q4 = most Zbtb7a-dependent). Zbtb7a-dependent DHS levels are expressed as the differences (left panel) or percentage reduction (right panel) in mean cut frequencies within ±600 bp surrounding the TSS, compared to Zbtb7a-knockdown fibroblasts. Lines in boxplots indicate median values; whiskers extend to the most extreme data within 1.5× the IQR from the box; outliers are not shown. Significance of difference in Zbtb7a dependence of DHS between least (Q1) and most (Q4) Zbtb7a-dependent promoters *P =* 2.3 × 10^−14^. Additional details of statistical analysis are provided as Supporting information, and numerical values underlying figures are reported in S1 Data. ChIP-seq, chromatin immunoprecipitation sequencing; IQR, interquartile range; TSS, transcription start site.
(PDF)

S5 Fig Endogenous mRNA expression levels of target genes of putative Zbtb7a-utilising TFs, in control (congenic p53ko, black) and Zbtb7a-knockout (grey) fibroblasts, and in Zbtb7a-knockout fibroblasts with experimentally restored Zbtb7a expression (blue), measured by quantitative real-time PCR. Zbtb7a-knockout and congenic-control fibroblasts are both derived on a p53-knockout background, to prevent premature senescence [20]. mRNA levels are expressed relative to the level in unstimulated control cells. Error bars indicate SEM. (A) *Zbtb7a* mRNA levels, reflecting expression of both the endogenous *Zbtb7a* gene (in control cells) as well as that driven by the *Zbtb7a* cDNA transgene (in cells with restored Zbtb7a expression). (B) Expression of the control housekeeping genes *Taf1* and *Hprt*. (C–G) Expression of target genes of cJun (C), Tead2 (D), Runx2 (E), Cebpd (F), and NFκB (G) identified by microarray analysis (see Fig 4). Expression levels of genes that are induced by TNF-α treatment (including NFκB targets and the Cebpd target *Ptx3*) are shown separately for unstimulated cells and for cells stimulated by TNF-α treatment. Statistical analysis is provided as Supporting information, and numerical values underlying figures are reported in S1 Data. NFκB, nuclear factor kappa B; SEM, standard error of the mean; TNF-α, tumour necrosis factor alpha.
(PDF)

S6 Fig (A) Outline of cellular reconstitution system used to analyse functions of p65 variants under conditions of normal expression levels in fibroblasts. (B) Expression levels of p65 variants in reconstituted p65-knockout fibroblasts. Flow cytometry analysis of p65-knockout (upper left) and normal (lower left) fibroblasts, after intracellular staining using antibodies recognising the p65 DBD, which is present in all p65 variants used. Centre panel: before sorting, transduced fibroblasts express a range of p65 levels and include cells with strong p65 overexpression; right panels: after sorting for low levels of co-expressed Tomato protein, cells express each p65 variant protein at similar levels to that of endogenous p65 in normal fibroblasts. (C–E) Function-impairing mutations within p65 TA3, which disrupt its interaction with Zbtb7a, reduce p65 TA3-driven changes to target promoter accessibility. Panels C, D: induced DNase-I hypersensitivity at p65 target promoters in TNF-α-treated fibroblasts expressing p65 TA3 or the loss-of-function mutant form of p65 TA3 (“TA3 mutant”). DNase-I hypersensitivity levels are shown at individual promoters as the differences to the levels observed in non-reconstituted p65-knockout fibroblasts (C) or as the differences between fibroblasts reconstituted with p65 TA3 mutant and with p65 TA3 (D). (E) DNase-I hypersensitivity changes induced by p65 TA3 or p65 TA3 mutant, at distinct groups of promoters in TNF-α-treated fibroblasts. DHS differences represent the mean changes in cut site frequencies at within a range of ±600 bp surrounding the TSS, compared to non-reconstituted p65-knockout fibroblasts. Lines in boxplots indicate median values; whiskers extend to the most extreme data within 1.5× the IQR from the box; outliers are not shown. (F, G) GFP reporter expression from plasmids containing 1 kb promoter sequences from the TA3-responsive *Saa3* (F) and *Gem* (G) genes, in transfected p65-knockout fibroblasts expressing the p65 minimal TA3 region. Red bars in (F) represent expression from an *Saa3* promoter with a targeted mutation to disrupt the single consensus Zbtb7a binding motif. The cloned *Gem* promoter (G) does not contain a consensus Zbtb7a motif (there is no motif within the 1 kb sequence immediately upstream of the TSS, and the maximal Zbtb7a ChIP signal at the endogenous *Gem* locus coincides with a consensus motif in the first intron), and the reporter plasmid is appended with the Gal4-UAS. Blue bars in (G) represent artificial recruitment of Zbtb7a to the cloned promoter by expression of Zbtb7a fused to the DBD of Gal4. White bars in (G) represent cells expressing of the Gal4DBD alone as a control. Error bars indicate SEM. Statistical analysis is provided as Supporting information, and numerical values underlying figures are reported in S1 Data. ChIP-seq, chromatin immunoprecipitation sequencing; DBD, DNA-binding domain; IQR, interquartile range; TNF-α, tumour necrosis factor alpha; TSS, transcription start site.
(PDF)

S7 Fig (A, B) Genome browser example tracks of DNase-I hypersensitivity surrounding the promoters of the TA3-responsive *Camp* gene (A), the control non-NFκB-regulated *Hprt* gene (B), in p65-knockout fibroblasts (top), in p65-knockout fibroblasts reconstituted with p65 (middle), and in p65-knockout fibroblasts reconstituted with p65 and with simultaneous knockdown of Zbtb7a (bottom), after TNF-α treatment. Lower tracks indicate predicted Zbtb7a and p65 binding peaks, and RefSeq genes. (C, D) Induced DNase-I hypersensitivity at control promoters, in TNF-α-treated control or Zbtb7a-knockdown fibroblasts expressing p65 (controls for Fig 6C and 6D). DNase-I hypersensitivity levels are shown at individual promoters as the differences to the levels observed in non-reconstituted p65-knockout fibroblasts (C) or as the differences between Zbtb7a knockdown and control fibroblasts (D). (E) Mean p65-induced DNase-I hypersensitivity levels across p65 target (left) or control (right) promoters, in TNF-α-treated control and Zbtb7a-knockdown fibroblasts. NFκB, nuclear factor kappa B; RefSeq, NCBI reference sequence database; TNF-α, tumour necrosis factor alpha.
(PDF)

S1 Text Statistical analysis & annotation of main & supporting figures. (PDF)

## Abbreviations

Bcl6: B cell lymphoma 6
BCoR: Bcl6 corepressor
BiFC: bimolecular fluorescence complementation
CGI: CpG island
ChIP-seq: chromatin immunoprecipitation sequencing
FBS: fetal bovine serum
FDR: false discovery rate
FPKM: RNA-sequencing fragments per kilobase transcript per million reads
GC: guanine-cytosine
GFP: green fluorescent protein
GMFI: geometric mean fluorescence intensity
GO: gene ontology
GST: glutathione S-transferase
HEK-293: human embryonic kidney cells 293
IQR: interquartile range
MS: mass spectrometry
NFκB: nuclear factor kappa B
NCoR: nuclear receptor corepressor
NuRD: nucleosome remodeling and deacetylase
POZ: pox virus zinc finger
RefSeq: NCBI reference sequence database
SEM: standard error of the mean
shRNA: short hairpin RNA
SILAC: stable isotope labelling of amino acids in cell culture
SMRT: silencing mediator for retinoid and thyroid receptors
TF: transcription factor
TNF-α: tumour necrosis factor alpha
TSS: transcription start site
ZF: zinc finger
