# Supplementary figures and images for: Zbtb7a is a transducer for the control of promoter accessibility by NF-kappa B and multiple other transcription factors
Source: PLoS Biol. 2018 May 29;16(5):e2004526. doi: 10.1371/journal.pbio.2004526 (PMC5993293; doi:10.1371/journal.pbio.2004526)

Figure S1

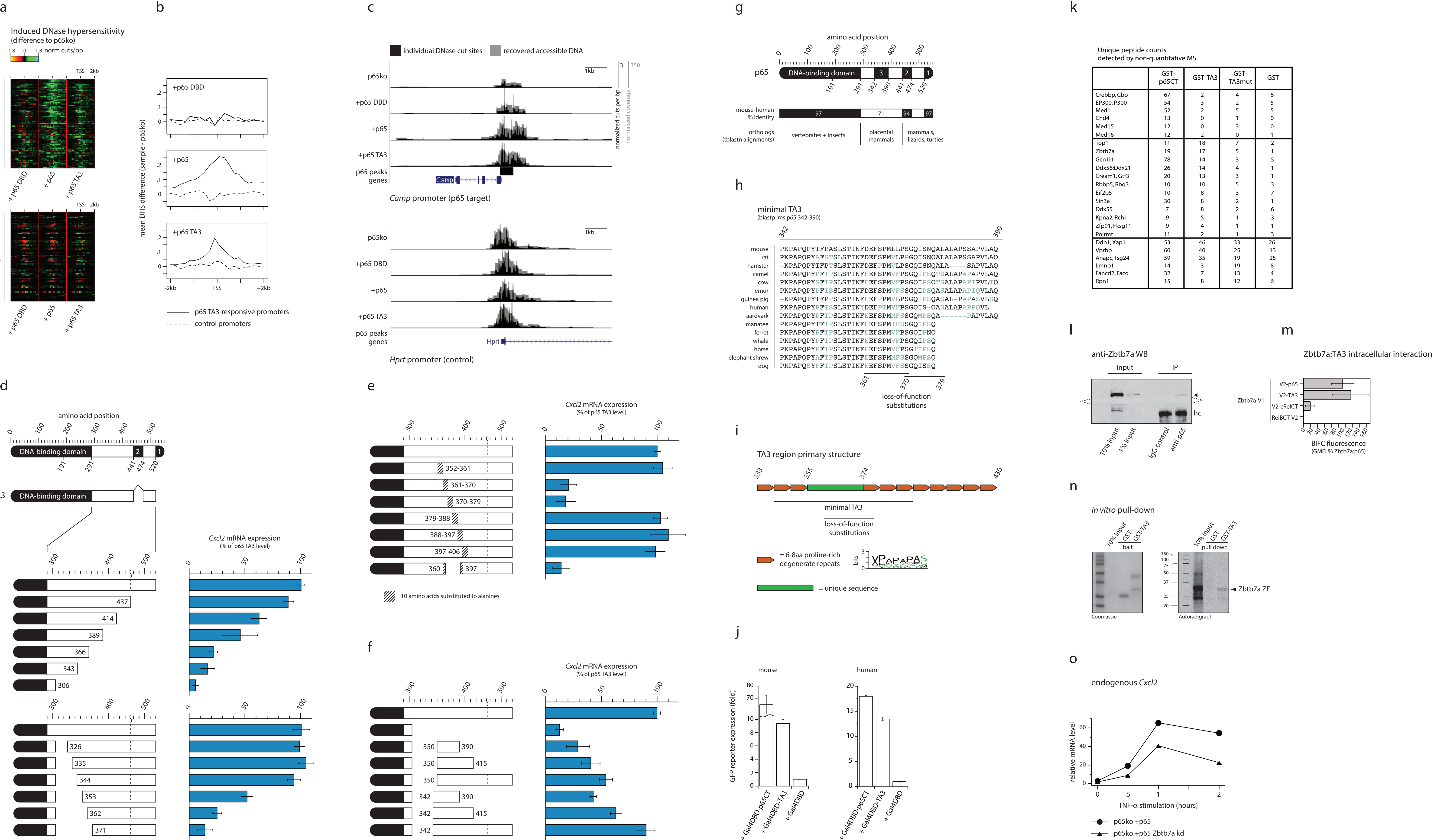

Supplement: S1 Fig — (A, B) DNase-I hypersensitivity levels induced by p65 DBD, p65 or by p65 TA3, at TA3-responsive or control (non-NFκB target) promoters, in TNF-α-treated p65-knockout fibroblasts. DNase-I hypersensitivity levels represent the mean normalised cut frequencies in 200 bp windows and are shown as the differences to the levels observed in p65-knockout fibroblasts, at individual promoters (A), or the mean levels across all promoters in each group (B). (C) Genome browser example tracks of DNase-I hypersensitivity surrounding the promoters of the TA3-responsive Camp gene (top), or the control non-NFκB target Hprt gene (bottom), in TNF-α-treated p65-knockout fibroblasts expressing p65 variants. Grey signal indicates coverage of recovered DNA fragments; black signal indicates the density of individual DNase-I cut sites. Lower tracks indicate predicted p65 binding peaks and RefSeq genes. (D–F) Identification of functional TA3 subregions. Variants of p65 TA3 were used to reconstitute p65-knockout fibroblasts and assayed by their ability to activate mRNA expression of the endogenous, TA3-responsive Cxcl2 gene after treatment with TNF-α. Numbers indicate the amino acid positions in full-length mouse p65. (D) Truncations and deletions of p65 TA3 indicate that removals of regions overlapping positions 344–389 result in >50% loss of activity. (E) Replacement of contiguous 10 amino acid regions within p65 TA3 with alanine residues indicates that positions 361–370 and 370–379 are each required for TA3 activity. The replacement mutant 361–370 >10A (third bar) was used as a loss-of-function mutant in the remainder of this study. (F) Expression of subregions of p65 TA3 indicate that the region spanning positions 342–390 (sixth bar) is sufficient to confer 43% of TA3 activity (and up to 50% activity in independent experiments; not shown). Inclusion of additional adjacent downstream regions (342–415; seventh bar) is able to further augment TA3 activity. We refer to positions 342–390 of p65 a [file pbio.2004526.s003.pdf]

Figure S2

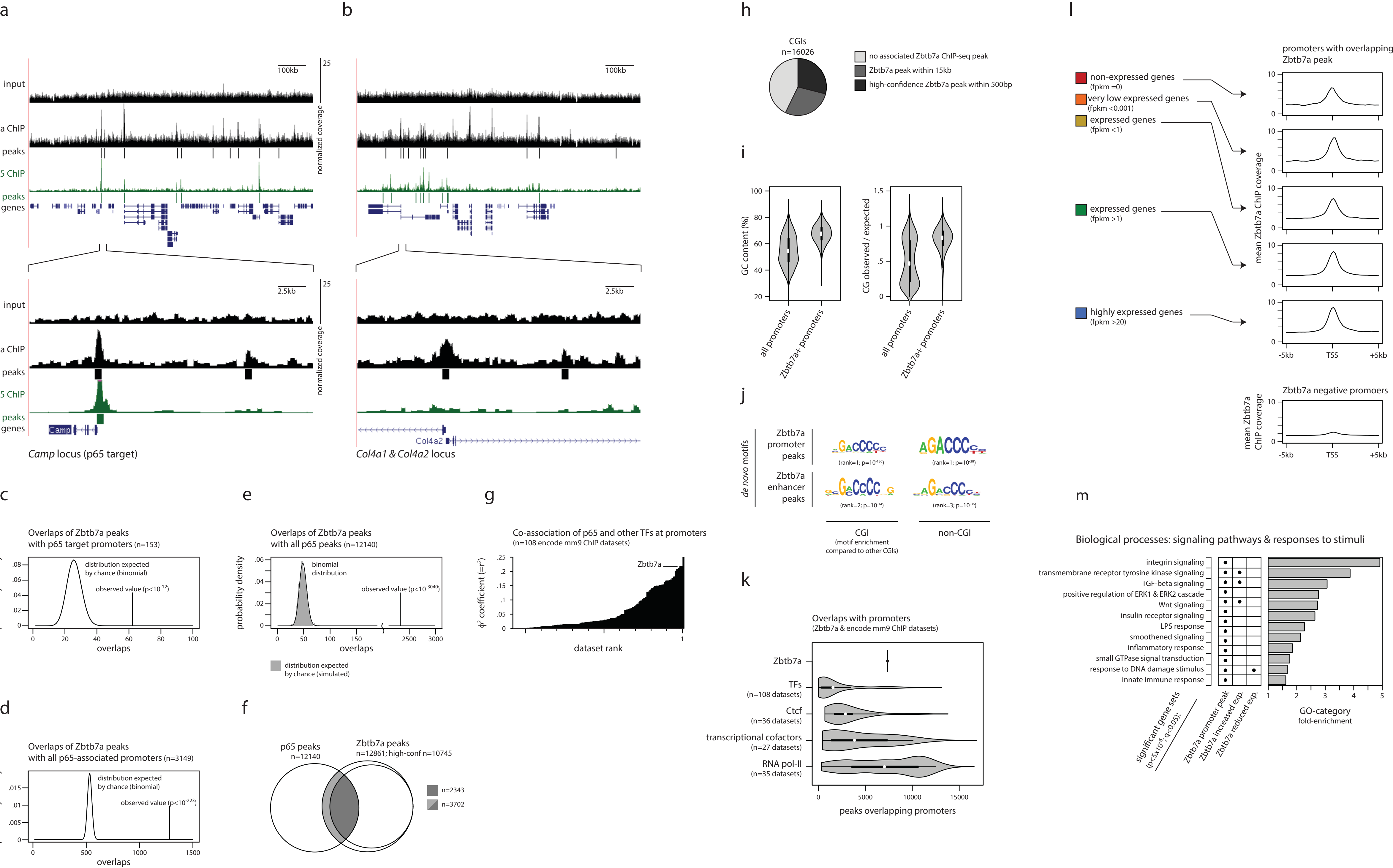

Supplement: S2 Fig — (A, B) Genome browser example tracks across the same 1 Mb and 25 kb genomic intervals shown in Fig 2 including the Camp (A) and Col4a1/2 (B) loci, indicating the p65 ChIP signal and locations of predicted p65 binding peaks (green). (C–F) Overlaps of Zbtb7a ChIP-seq peaks with p65 target promoters and ChIP-seq peaks. Number of overlaps between Zbtb7a ChIP-seq peaks and p65 target promoters ± 500 bp (C), all p65-associated promoters ± 500 bp (including many promoters without evidence for regulation of gene expression by p65 in fibroblasts, D) and all genome-wide p65 ChIP-seq peaks (E, F). Curves indicate the distribution of overlaps expected by chance (using a binomial model); grey bars (in panel E) indicate empirically determined distribution of overlaps, using 900 randomisations of the 2 peak datasets retaining interpeak distances; line indicates the observed number of overlaps and calculated P value. (G) Co-association of p65 and other TFs at promoters ± 500 bp. The ϕ2 statistic (identical to Pearson’s r2 correlation applied to binary data) is indicated for pairwise comparisons of overlaps at promoters of p65 and each of 108 encode ChIP-seq datasets for TFs and other factors that are available for mouse genome assembly mm9, plus the set of Zbtb7a peaks from this study. The co-association of p65 with Zbtb7a ranks fourth among all 109 datasets analysed. Similar results were obtained using the Jaccard statistic to quantify co-association (Zbtb7a ranks fifth among all 109 datasets). Note, however, that encode datasets are derived from diverse cell types, which may contribute to reduced data overlap. (H, I) Genomic context of Zbtb7a peaks. (H) Fraction of CGIs that are associated with predicted Zbtb7a ChIP-seq peaks. (I) GC content (left) and ratio of observed/expected CG dinucleotides (right) at all promoters ± 250 bp and at Zbtb7a-associated promoters. (J) The Zbtb7a binding motif is found at both CGI- and non-CGI-associated Zbtb7a ChIP-seq peaks. DNA sequence motifs [file pbio.2004526.s004.pdf]

Figure S3

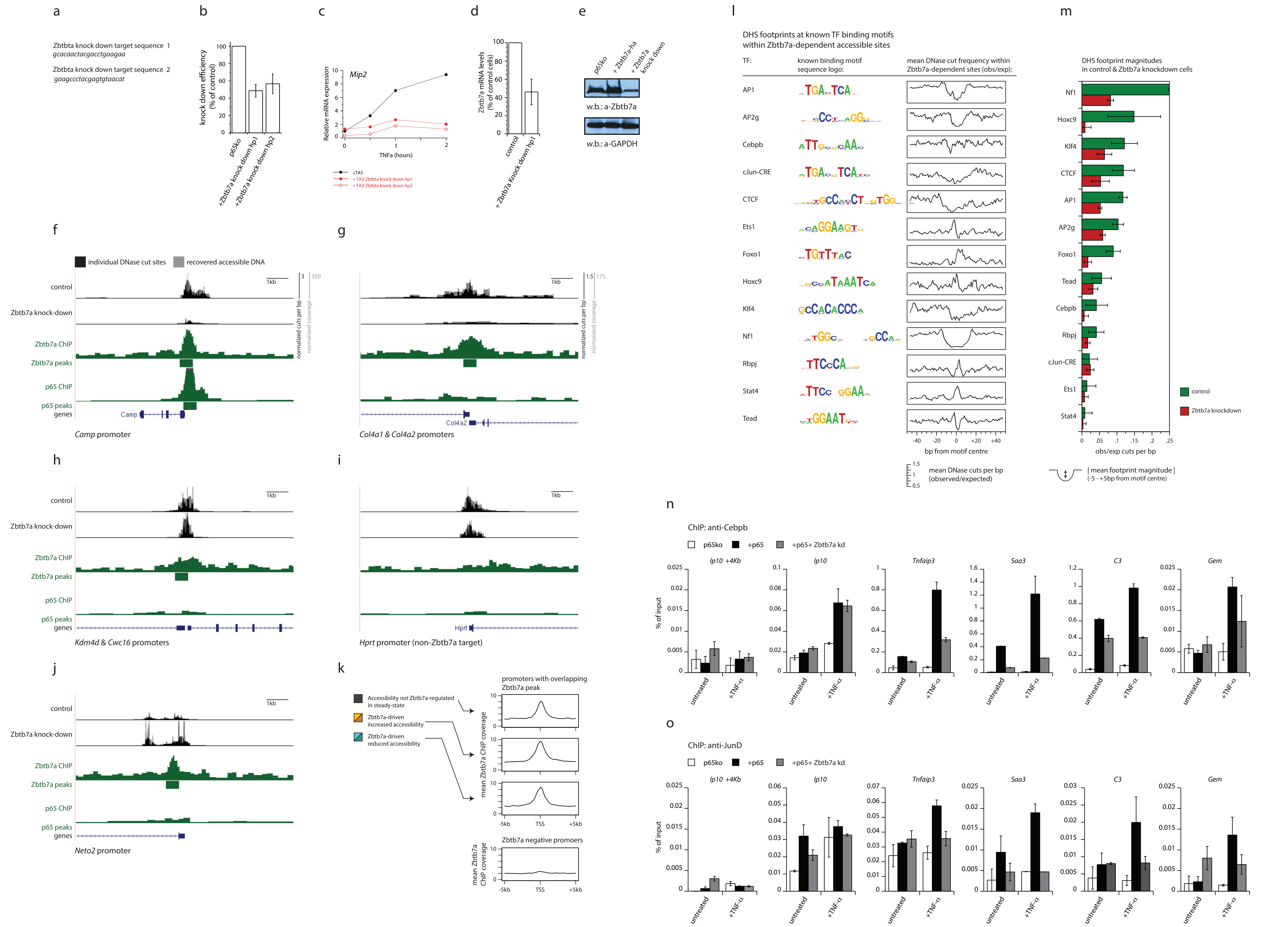

Supplement: S3 Fig — (A) Target sequences for knockdown hairpins within the Zbtb7a coding region. (B) Efficiency of stable mRNA knockdown by hairpins targeting Zbtb7a in p65-knockout fibroblasts, assayed by expression of GFP coded by an independent mRNA engineered to contain the same Zbtb7a-derived target sequences. Fibroblast cell lines with higher levels of knockdown (up to 90%) could be generated, but they exhibited reduced proliferation and could not be efficiently expanded (in agreement with the senescence described in Zbtb7a-knockout fibroblasts [20]). Error bars indicate SEM. (C) Effect of independent Zbtb7a-knockdown hairpins on TNF-α-induced expression of the TA3-responsive p65 target gene Cxcl2, in fibroblasts expressing p65 TA3. In parallel control experiments using hairpins directed against Gfp, Cxcl2 expression levels were unchanged (not shown). Based on the consistency of the inhibition of Cxcl2 mRNA expression mediated by both hairpins, we used hairpin 1 for all subsequent experiments in this study. (D) Endogenous Zbtb7a mRNA levels in p65-knockout fibroblasts after stable knockdown of Zbtb7a, assayed by quantitative PCR. Error bars indicate SEM. (E) Zbtb7a protein levels in p65-knockout fibroblasts (left), upon ectopic expression of an epitope-tagged form of Zbtb7a (centre), or after stable knockdown of Zbtb7a (right), assayed by immunoblotting for Zbtb7a (top), or Gapdh as a control (bottom). Quantitation of the immunoblot signal indicates that Zbtb7a protein levels are depleted by around 70% to 80% in knockdown cells. (F–J) Genome browser example tracks of DNase-I hypersensitivity (F–I) across the same genomic loci shown in Fig 3 and (J) surrounding the Neto2 gene promoter (Zbtb7a-bound and Zbtb7a-dependent reduced accessibility), in control or Zbtb7a-knockdown fibroblasts, and indicating the Zbtb7a (black) and p65 (green) ChIP signals and the locations of predicted Zbtb7a- and p65 binding peaks. (K) Zbtb7a-associated promoters that do or do not exhibit ongoing regulat [file pbio.2004526.s005.pdf]

Figure S4

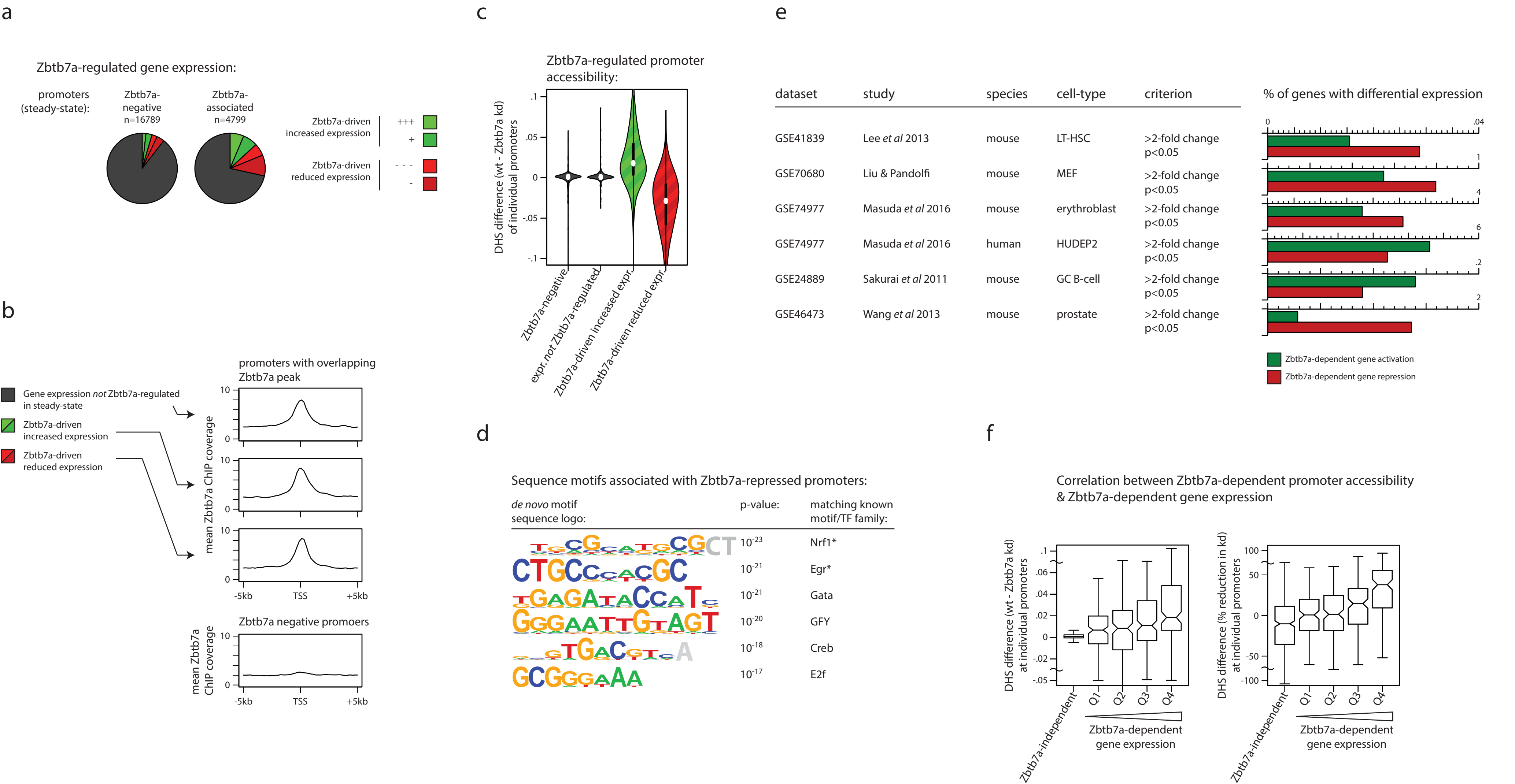

Supplement: S4 Fig — (A) Fractions of promoters of genes with changed mRNA expression in Zbtb7a-knockdown fibroblasts. Left: promoters without any associated Zbtb7a peak (“Zbtb7a-negative”); right: promoters with associated Zbtb7a peaks. Green/red slices indicate promoters with increased/reduced mRNA expression in control fibroblasts compared to Zbtb7a-knockdown fibroblasts, at P < 0.05 and with an affymetrix signal difference of ≥0.5 (indicated as “+++” / “−−−”) or ≥0.2 (indicated as “+” / “−”). (B) Zbtb7a-associated promoters that do or do not exhibit ongoing regulation of expression by Zbtb7a in fibroblasts display comparable levels of Zbtb7a. Mean Zbtb7a ChIP signals across promoters with overlapping Zbtb7a ChIP-seq peaks (upper panels) and at promoters without any overlapping predicted Zbtb7a peak (bottom panel). Promoters are divided according to whether or not they exhibit Zbtb7a-driven increased or decreased gene expression, as in panel A. (C) Zbtb7a dependence of DNase-I hypersensitivity levels at Zbtb7a-negative promoters and at Zbtb7a-associated promoters of genes with expression that is not Zbtb7a regulated, or with Zbtb7a-driven increased or reduced expression. Zbtb7a-dependent DHS levels represent differences in mean cut frequencies within ±600 bp surrounding the TSS, compared to Zbtb7a-knockdown fibroblasts. Dots in violins indicate mean values. (D) DNA sequence motifs that are enriched at Zbtb7a-repressed promoters (defined as genes with increased expression in Zbtb7a-knockdown fibroblasts). Motif enrichment analysis was performed separately using all promoters, Zbtb7a-independent promoters, and Zbtb7a-dependent promoters as background sets, and motifs exhibiting consistent enrichment are shown. Known TF families with specificities matching each motif are indicated; additional letters of matching known motifs that are not present in the identified de novo motif logos are indicated in grey. *Note that the 2 most enriched motifs (resembling the specificities of the Nrf1 an [file pbio.2004526.s006.pdf]

Figure S5

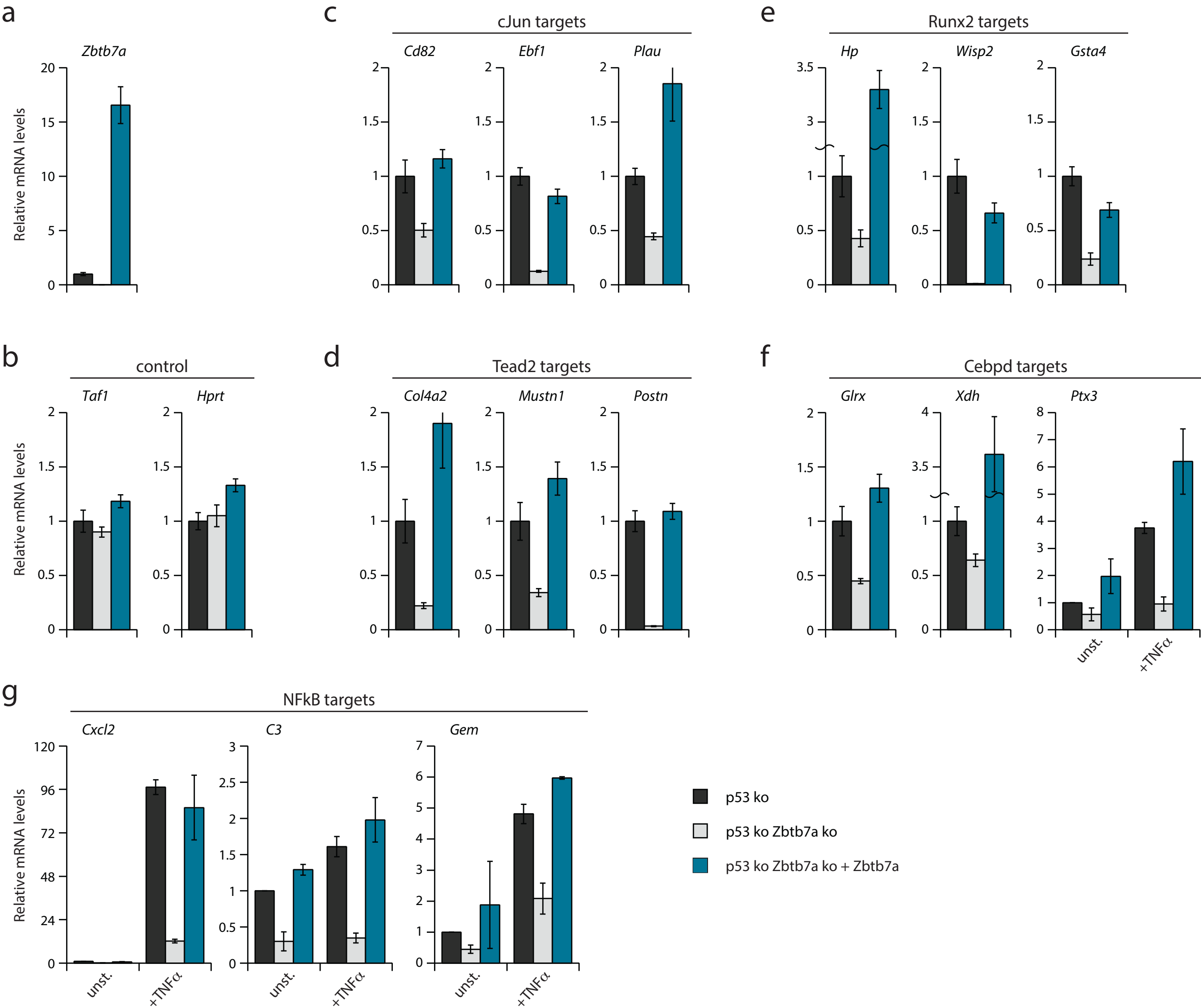

Supplement: S5 Fig — Zbtb7a-knockout and congenic-control fibroblasts are both derived on a p53-knockout background, to prevent premature senescence [20]. mRNA levels are expressed relative to the level in unstimulated control cells. Error bars indicate SEM. (A) Zbtb7a mRNA levels, reflecting expression of both the endogenous Zbtb7a gene (in control cells) as well as that driven by the Zbtb7a cDNA transgene (in cells with restored Zbtb7a expression). (B) Expression of the control housekeeping genes Taf1 and Hprt. (C–G) Expression of target genes of cJun (C), Tead2 (D), Runx2 (E), Cebpd (F), and NFκB (G) identified by microarray analysis (see Fig 4). Expression levels of genes that are induced by TNF-α treatment (including NFκB targets and the Cebpd target Ptx3) are shown separately for unstimulated cells and for cells stimulated by TNF-α treatment. Statistical analysis is provided as Supporting information, and numerical values underlying figures are reported in S1 Data. NFκB, nuclear factor kappa B; SEM, standard error of the mean; TNF-α, tumour necrosis factor alpha. (PDF) [file pbio.2004526.s007.pdf]

Figure S6

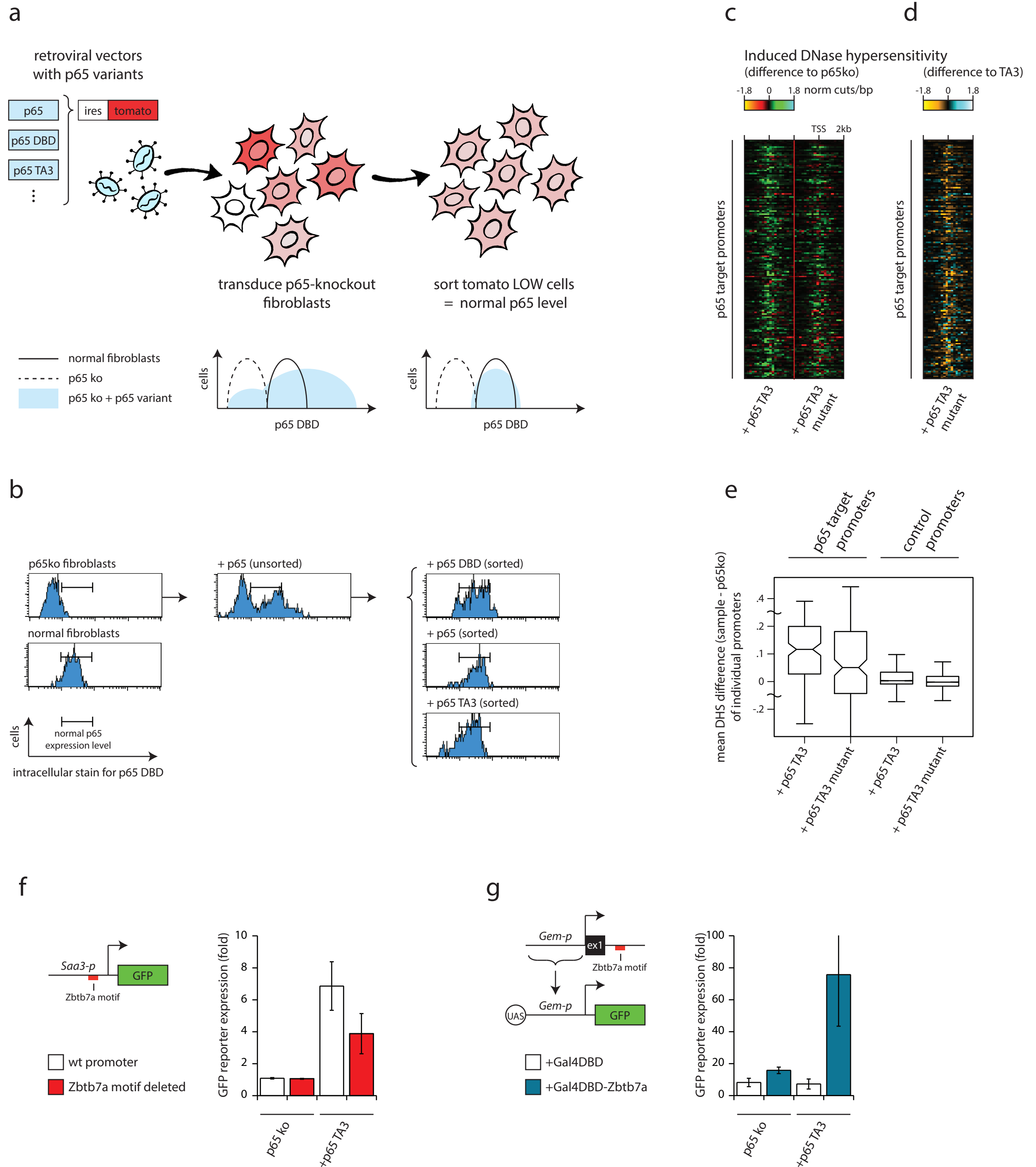

Supplement: S6 Fig — (A) Outline of cellular reconstitution system used to analyse functions of p65 variants under conditions of normal expression levels in fibroblasts. (B) Expression levels of p65 variants in reconstituted p65-knockout fibroblasts. Flow cytometry analysis of p65-knockout (upper left) and normal (lower left) fibroblasts, after intracellular staining using antibodies recognising the p65 DBD, which is present in all p65 variants used. Centre panel: before sorting, transduced fibroblasts express a range of p65 levels and include cells with strong p65 overexpression; right panels: after sorting for low levels of co-expressed Tomato protein, cells express each p65 variant protein at similar levels to that of endogenous p65 in normal fibroblasts. (C–E) Function-impairing mutations within p65 TA3, which disrupt its interaction with Zbtb7a, reduce p65 TA3-driven changes to target promoter accessibility. Panels C, D: induced DNase-I hypersensitivity at p65 target promoters in TNF-α-treated fibroblasts expressing p65 TA3 or the loss-of-function mutant form of p65 TA3 (“TA3 mutant”). DNase-I hypersensitivity levels are shown at individual promoters as the differences to the levels observed in non-reconstituted p65-knockout fibroblasts (C) or as the differences between fibroblasts reconstituted with p65 TA3 mutant and with p65 TA3 (D). (E) DNase-I hypersensitivity changes induced by p65 TA3 or p65 TA3 mutant, at distinct groups of promoters in TNF-α-treated fibroblasts. DHS differences represent the mean changes in cut site frequencies at within a range of ±600 bp surrounding the TSS, compared to non-reconstituted p65-knockout fibroblasts. Lines in boxplots indicate median values; whiskers extend to the most extreme data within 1.5× the IQR from the box; outliers are not shown. (F, G) GFP reporter expression from plasmids containing 1 kb promoter sequences from the TA3-responsive Saa3 (F) and Gem (G) genes, in transfected p65-knockout fibroblasts expressing the p65 minimal TA3 reg [file pbio.2004526.s008.pdf]

Figure S7

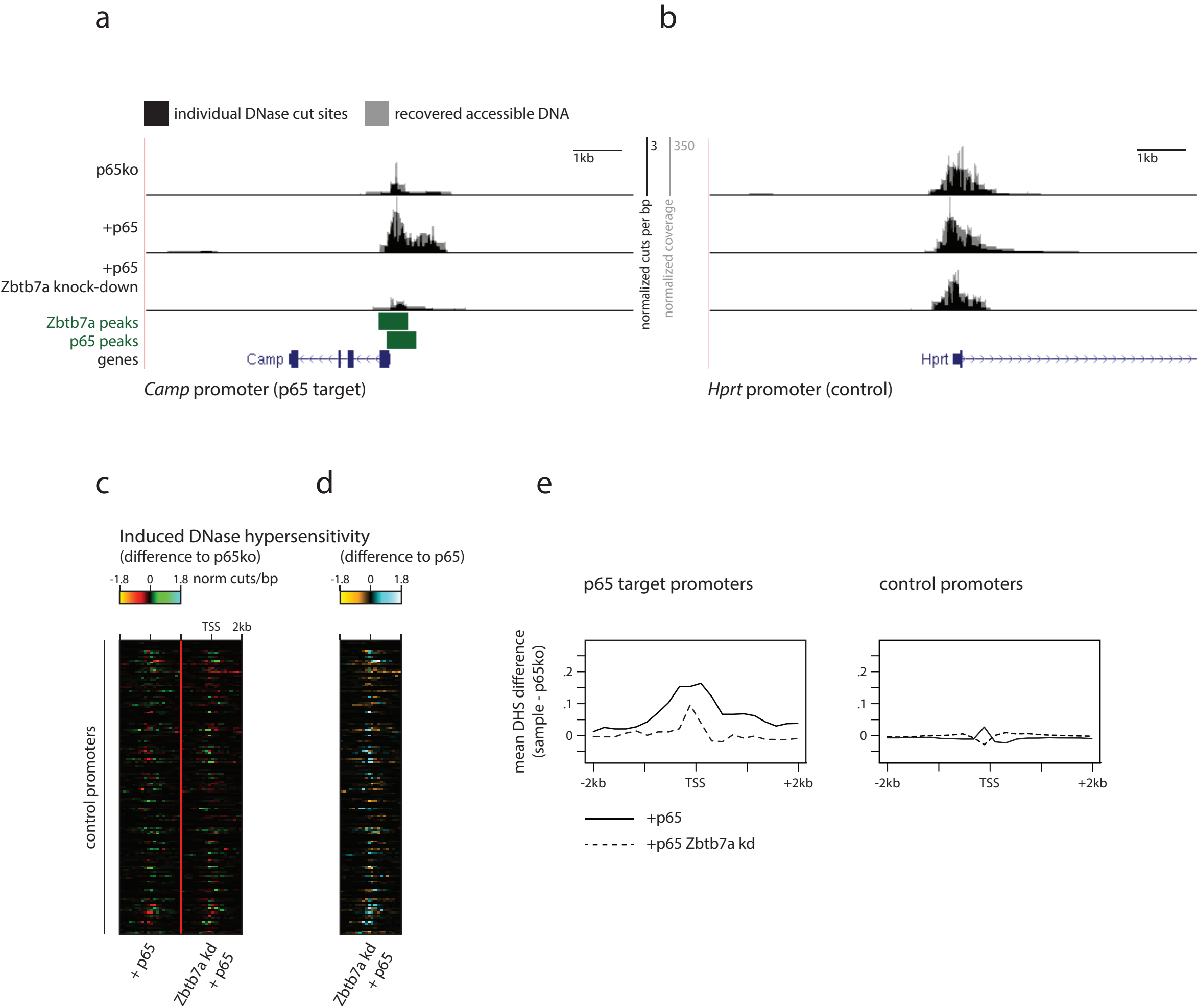

Supplement: S7 Fig — (A, B) Genome browser example tracks of DNase-I hypersensitivity surrounding the promoters of the TA3-responsive Camp gene (A), the control non-NFκB-regulated Hprt gene (B), in p65-knockout fibroblasts (top), in p65-knockout fibroblasts reconstituted with p65 (middle), and in p65-knockout fibroblasts reconstituted with p65 and with simultaneous knockdown of Zbtb7a (bottom), after TNF-α treatment. Lower tracks indicate predicted Zbtb7a and p65 binding peaks, and RefSeq genes. (C, D) Induced DNase-I hypersensitivity at control promoters, in TNF-α-treated control or Zbtb7a-knockdown fibroblasts expressing p65 (controls for Fig 6C and 6D). DNase-I hypersensitivity levels are shown at individual promoters as the differences to the levels observed in non-reconstituted p65-knockout fibroblasts (C) or as the differences between Zbtb7a knockdown and control fibroblasts (D). (E) Mean p65-induced DNase-I hypersensitivity levels across p65 target (left) or control (right) promoters, in TNF-α-treated control and Zbtb7a-knockdown fibroblasts. NFκB, nuclear factor kappa B; RefSeq, NCBI reference sequence database; TNF-α, tumour necrosis factor alpha. (PDF) [file pbio.2004526.s009.pdf]
