# Supplementary material for: Zbtb7a is a transducer for the control of promoter accessibility by NF-kappa B and multiple other transcription factors
Source: PLoS Biol. 2018 May 29;16(5):e2004526. doi: 10.1371/journal.pbio.2004526 (PMC5993293; doi:10.1371/journal.pbio.2004526)
Supplement: S1 Text — (PDF) [file pbio.2004526.s010.pdf]

Zbtb7a is a transducer for the control of promoter accessibility by NF-kappa B and multiple other transcription factors

José Miguel Ramos Pittol, Agata Oruba, Gerhard Mittler, Simona Saccani and Dominic van Essen

Statistical analysis & annotation of main & supporting figures

Sample sizes in all experiments were not selected to detect any pre-specified effect size(s). All samples were included in analyses unless otherwise specified.

Fig 1

1B - Experiments shown are representative of 3 experiments using independently grown cell cultures; error bars indicate s.e.m of replicate transfections within each experiment (sample size=3). Significances of reporter expression levels driven by p65 variants *vs* untransduced p65ko cells: NFkB motifs only +p65 TA1&2 $p=3.6x10^{-6}$, +p65 TA3 p=0.54 (two-tailed Student's t-test without assumption of equal variance).

1C - The results shown are representative of more than 5 experiments using independently grown cell cultures, with similar results.

1D - Significances of induced DHS differences: TA3-responsive promoters *vs* control promoters +p65 $p=9.4x10^{-24}$, +p65 TA3 $3.8x10^{-24}$ (two-tailed Mann-Whitney u-test; no assumption of equal distribution or variance).

1E - Experiments shown are representative of 3 experiments using independently grown cell cultures; error bars indicate s.e.m of replicate transfections within each experiment (sample size=3). Significances of reporter expression levels driven by p65 variants *vs* untransduced p65ko cells: *Cxcl2* promoter +p65 TA1&2 $p=5.2x10^{-13}$, +p65 TA3 $p=9.6x10^{-8}$; *Saa3* promoter +p65 TA1&2 $p<10^{-15}$, +p65 TA3 $p<10^{-15}$ (two-tailed Student's t-test without assumption of equal variance).

1F - The results shown are representative of more than 5 experiments using independently grown cell cultures, with similar results; error bars indicate s.e.m of replicate quantitative PCRs within one experiment (sample size=3).

1H - The indicated 'combined SILAC ratio' is the mean SILAC ratio for each protein detected in two independent SILAC pull-down experiments, in which 'heavy' and 'light' labelled nuclear extracts were exchanged for each bait protein (considered as 'forward' and 'reverse' pull-downs). Ratios of proteins detected in only one pull-down direction were not considered (since these may be affected by differences in nuclear extract preparation). Mean unique peptides detected for each protein: 10.9 (sd 13.8) forward, 11.6 (sd 15.6) reverse. Significance of Zbtb7a SILAC ratio (after transformation of all less-extreme ratios to normal distribution) $p=1.7x10^{-3}$.

1I - Pull-down blots were performed in 2 independent experiments, with similar results.

1J - The results shown are representative of 4 independent BiFC experiments, with similar results; error bars indicate s.e.m of replicate transfections within one experiment (sample size=3).

1K - The results shown are representative of more than 5 experiments using independently grown cell cultures, with similar results.

Fig 2

Zbtb7a ChIP-seq was performed on a total of 9 samples in 2 separate experiments using independently grown cell cultures, all with similar results: coverage across all

predicted Zbtb7a peaks was highly correlated between all datasets (mean Pearson's r =0.92 for all pairwise comparisons [range 0.86-0.95]).

2C - Zbtb7a peaks analysed n=12861, promoters n=23270, enhancers (identified as H3K4me1 peaks in fibroblasts) n=81778. Enrichments of promoters and enhancers among Zbtb7a peaks are both significant with $p<10^{-1000}$ (two-tailed binomial test).

2D - Group sizes for data analysis: all promoter regions n=23270, p65 target promoter regions n=153. Enrichments of Zbtb7a peaks associated with all promoter regions, and with p65 target promoter regions, are both significant with $p<10^{-1000}$ (two-tailed binomial test).

2E - Group sizes for data analysis: all Zbtb7a peaks n=10743, Zbtb7a promoter peaks n=5278. P-values for motif enrichment were calculated using the Homer software package, based on an expected binomial distribution of motifs within background sequences matched for mono-, di- and tri-nucleotide content.

2F - Number of promoters with overlapping Zbtb7a peak: non-expressed genes (fpkm=0) n=253 (out of 8393), very low expressed genes (0<fpkm<0.001) n=281 (out of 1197), expressed genes (0.001≤fpkm<1) n=776 (out of 5062), expressed genes (1≤fpkm<20) n=2902 (out of 8245), highly expressed genes (20≤fpkm) n=1134 (out of 2984). Number of Zbtb7a-negative promoters analysed n=7984.

2G - Group sizes for data analysis: GO-annotated promoters n=24203, Zbtb7a-peak annotated promoters n=5770, Zbtb7a-up annotated promoters n=330, Zbtb7a-down annotated promoters n-299, total GO annotation terms of all promoters n=16613. Significantly enriched GO terms are calculated using two-tailed binomial tests with Holm-Bonferroni correction for multiple testing. Corrected q-values: cell migration (GO:0016477) $q=8.4x10^{-11}$; positive reg. of cell proliferation (GO:0008284) $q=9.8x10^{-8}$; negative reg. of transcription (GO:0045892) $q<10^{-12}$; protein phosphorylation (GO:0006468) $q<10^{-12}$; signal transduction (GO:0035556) $q=3.8x10^{-10}$; positive reg. of transcription (GO:0045893) $q<10^{-12}$; negative reg. of cell proliferation (GO:0008285) $q<10^{-12}$; chromatin modification (GO:0016568) $q=2.9x10^{-8}$; inflammatory response (GO:0006954) $q=1.4x10^{-4}$; apoptotic process (GO:0006915) $q=1.9x10^{-11}$; multicellular organismal development (GO:0007275) $q=7.4x10^{-7}$; response to DNA damage stimulus (GO:0006974) $q=5.3x10^{-7}$.

Fig 3

DHS-seq was performed in 2 separate experiments using independently grown cell cultures, with similar results.

3E - Significance of enrichment for Zbtb7a-regulated accessibility among promoters associated with Zbtb7a peaks, compared to Zbtb7a-negative promoters: $p=1.2x10^{-957}$ (two-tailed binomial test).

3F - Significance of enrichment for Zbtb7a-regulated accessibility among enhancers associated with Zbtb7a peaks, compared to Zbtb7a-negative enhancers: $p=4.8x10^{-294}$ (two-tailed binomial test).

3G - Significance of enrichment for Zbtb7a-regulated accessibility among TA3-responsive p65 target promoters, compared to Zbtb7a-negative promoters: $p=2.1x10^{-39}$ (two-tailed binomial test).

Fig 4

4A - *De novo* motif enrichment analysis surrounding Zbtb7a peaks was performed independently using either nucleotide-content-matched random background regions or CpG-island (CGI)-containing regions (to control for the enrichment of CGIs at Zbtb7a peaks); the motifs shown in Fig 4A (or closely-matching motifs) were identified in both analyses. P-values for motif enrichment were calculated using the

Homer software package, based on an expected binomial distribution of motifs within background sequences.

4C - Group sizes for data analysis: all promoters n=27946; Runx2-, cJun- Tead2- & Cepbd targets n=200 each; p65 direct targets n=153. Significances of enrichment for promoters with Zbtb7a-regulated accessibility are corrected for multiple testing of TF family members where applicable: Runx2 $q=1.4x10^{-6}$, cJun $p=1.4x10^{-6}$, Tead2 $q=2.3x10^{-11}$, Cebpd $q=1.7x10^{-8}$, p65 $p=1.9x10^{-29}$ (two-tailed binomial test with Bonferroni correction).

4D - Microarray analysis of Zbtb7a knock-down cells was performed on 2 independent sets of shRNA-transduced cells. Group sizes for data analysis: control genes with Zbtb7a-regulated accessibility n=400; Runx2-, cJun- Tead2- & Cepbd targets n=200 each; p65 direct targets n=52. Significances of mRNA expression differences *vs* control genes: Runx2 targets $p=8.9x10^{-3}$; cJun targets $p=2.3x10^{-7}$; Tead2 targets $p=9.7x10^{-11}$; Cebpd targets $p=4.9x10^{-6}$; p65 targets $p=1.8x10^{-14}$ (two-tailed Mann-Whitney u-test; no assumption of equal distribution or variance).

<u>Fig 5</u>

5A - Microarray analysis was performed on 3 independent sets of transduced cells for each experimental condition. Gene group sizes for data analysis: p65 target promoters n=153; TA3-responsive genes n=73; non-TA3-responsive genes n=80; matched control genes n=7189.

5B - Significances of Zbtb7a-dependence of TA3-responsive genes *vs* control genes: $p=3.0x10^{-14}$ (two-tailed Mann-Whitney u-test; no assumption of equal distribution or variance).

5C - The experiment shown is representative of 2 experiments using independently grown cell cultures; error bars indicate s.e.m of replicate transfections within each experiment (sample size=3). Significances of reporter expression levels driven by Gal4DBD-TA3 *vs* Gal4DBD: wt fibroblasts $p=8.1x10^{-7}$, Zbtb7a-knockout fibroblasts not significant (p=0.23) (two-tailed Student's t-test without assumption of equal variance).

5E - Significances of DHS differences at non TA3-responsive promoters: +p65 TA3 *vs* Zbtb7a kd $p=4.7x10^{-2}$, *vs* control promoters $p=1.5x10^{-2}$ (two-tailed Mann-Whitney u-test; no assumption of equal distribution or variance).

5F - DHS-seq using the mutant form of TA3 was performed in 2 separate experiments using independently grown cell cultures, with similar results.

<u>Fig 6</u>

6A - Zbtb7a ChIP-seq was performed on a total of 9 samples in 2 separate experiments (including 3 samples in p65-knockout fibroblasts) using independently grown cell cultures, all with similar results. Zbtb7a ChIP coverage is less than 2-fold changed in p65-knockout cells at 147 of 153 NFkB target promoters in untreated cells (> 2-fold Zbtb7a loss at 4 promoters, gain at 2 promoters); and at 150 NFkB target promoters in TNF-α-treated cells (>2-fold loss at 1 promoter, gain at 2 promoters).

6B - p65 ChIP-seq was performed on a total of 9 samples in 3 separate experiments (including 2 samples in Zbtb7a-knockout fibroblasts) using independently grown cell cultures, with similar results. p65 ChIP coverage is less than 2-fold changed in Zbtb7a-knockout cells at 141 of 153 NFkB target promoters in untreated cells (> 2-fold p65 loss at 1 promoter, gain at 11 promoters); and at 135 NFkB target promoters in TNF-α-treated cells (>2-fold loss at 10 promoters, gain at 8 promoters).

6E - Significances of induced DHS differences at p65 target promoters +p65 *vs* Zbtb7a kd +p65: $p=2.4x10^{-8}$; at p65 target promoters *vs* control promoters: $p=1.5x10^{-38}$ (two-tailed Mann-Whitney u-test; no assumption of equal distribution or variance).

6F - Significances of induced DHS differences at p65-binding enhancers +p65 *vs* Zbtb7a kd +p65: $p=3.6x10^{-36}$, at p65-binding enhancers *vs* control enhancers: $p=1.1x10^{-134}$ (two-tailed Mann-Whitney u-test; no assumption of equal distribution or variance).

6G - Significances of differences *vs* control genes: TA3-responsive +p65 $p=1.6x10^{-26}$; non-TA3-responsive +p65 $p=3.6x10^{-38}$ (two-tailed Mann-Whitney u-test; no assumption of equal distribution or variance).

6H - Significances of TA3-dependence of gene expression levels for each group: TA3-responsive *vs* control genes $p=2.3x10^{-12}$, non-TA3-responsive *vs* control genes $p=1.0x10^{-4}$, TA3-responsive *vs* non-TA3-responsive genes $p=1.3x10^{-3}$ (two-tailed Mann-Whitney u-test; no assumption of equal distribution or variance). TA3-dependent genes (defined as those with log2 Affymetrix expression scores p65 - p65 TA1&2 > 0.8) within each group: all p65 targets 50/153 (32%), TA3-responsive genes 36/73 (49%), non-TA3-responsive genes 14/80 (17%), control genes 67/7189 (0.9%); significance of TA3-responsive *vs* non-TA3-responsive $p=5.9x10^{-5}$ (chi-squared test on 2x2 table; $\chi^2=16.1$, degrees of freedom=1).

Fig S1

S1D-F - The results shown are representative of 2 (d,e) or 3 (f) experiments using independently grown cell cultures; error bars indicate s.e.m of replicate transfections within each experiment (sample size=3).

S1J - The results shown are representative of 4 (mouse cells; including experiment in Fig 6C) or 2 (human cells) experiments using independently grown cell cultures; error bars indicate s.e.m of replicate transfections within each experiment (sample size=3). Significances of reporter expression levels driven by Gal4DBD-TA3 *vs* Gal4DBD: mouse fibroblasts $p=6.7x10^{-54}$, human HEK-293 cells $2.8x10^{-261}$ (two-tailed Student's t-test without assumption of equal variance).

S1L - The results shown are representative of 2 independent immunoprecipitation experiments, with similar results.

S1M - The results shown are representative of 3 independent BiFC experiments, with similar results; error bars indicate s.e.m of replicate transfections (sample size=3).

S1N - The results shown are representative of 2 independent pull-down experiments, with similar results.

S1O - The results shown are representative of more than 5 experiments using independently grown cell cultures, with similar results.

Fig S2

S2G - Group sizes for data analysis: p65 promoter peaks n=6055, Zbtb7a promoter peaks n=7363, encode ChIP-seq promoter peaks n=55-10141 (mean 3168, sd 2823).

S2H - CGIs n=16026. Enrichment of Zbtb7a peaks associated with CGIs are significant with $p<10^{-1000}$ (two-tailed binomial test).

S2I - Group sizes for data analysis: all promoters n= 28941, Zb-positive promoters n=4799. Significances of elevated GC content among Zbtb7a-positive promoters $p\approx10^{-1026}$, and of observed/expected CG ratios among Zbtb7a-positive promoters

$p \approx 10^{-818}$ (two-tailed Mann-Whitney u-test; no assumption of equal distribution or variance).

S2J - Group sizes for data analysis: Zbtb7a CGI promoter peaks n=3575, Zbtb7a non-CGI promoter peaks n=1145, Zbtb7a CGI enhancer peaks n=224, Zbtb7a non-CGI enhancer peaks n=1811. P-values for motif enrichment were calculated using the Homer software package, based on an expected binomial distribution of motifs within background sequences matched for mono-, di- and tri-nucleotide content either selected randomly (for non-CGI peaks) or within CGIs (for CGI peaks).

S2M - Significantly enriched GO terms are calculated using two-tailed binomial tests with Holm-Bonferroni correction for multiple testing. Corrected q-values: integrin signaling (GO:0007229) $q=3.1 \times 10^{-5}$; TM receptor tyrosine kinase activity (GO:0007169) $q=5.0 \times 10^{-4}$; TGF-beta signaling (GO:0007179) $q=1.3 \times 10^{-7}$; positive regulation of ERK1 & ERK2 cascade (GO:0070374) $q=2.0 \times 10^{-2}$; Wnt signaling (GO:0016055) $q=2.4 \times 10^{-9}$; insulin receptor signaling (GO:0008286) $q=1.2 \times 10^{-3}$; LPS response (GO:0071222) $q=3.2 \times 10^{-6}$; smoothened signaling (GO:0007224) $q=9.6 \times 10^{-3}$; inflammatory response (GO:0006954) $q=1.4 \times 10^{-4}$; small GTPase signal transduction (GO:0007264) $q=3.6 \times 10^{-3}$; response to DNA damage stimulus (GO:0006974) $q=5.3 \times 10^{-7}$; innate immune response (GO:0045087) $q=2.4 \times 10^{-2}$.

<u>Fig S3</u>

S3B,C - The results shown are representative of 3 knock-down experiments with both hairpins, each using independently grown cell cultures, with similar results. Error bars in panel b indicate s.e.m of replicate transductions within one experiment (sample size=3).

S3D - The results shown are representative of more than 5 knock-down experiments using independently grown cell cultures, with similar results. Error bar indicates s.e.m. of mRNA levels relative to those of control cells of replicate quantitative PCRs.

S3K - Number of promoters with overlapping Zbtb7a peak with accessibility *not* Zbtb7a-regulated n=3591, with Zbtb7a-driven increased accessibility n=214, with Zbtb7a-driven reduced accessibility n=246.

S3M - Number of analysed footprints within Zbtb7a-dependent sites and significances of differences in footprint magnitudes between control and Zbtb7a knockdown cells: Nf1 n=395, $p=4.0 \times 10^{-53}$; HoxC9 n=66, $p=1.5 \times 10^{-4}$; Klf4 n=134, $p=3.8 \times 10^{-3}$; CTCF n=52, $p=6.1 \times 10^{-4}$; AP1 n=1008, 6.0×10^{-24}; AP2g n=289, $p=2.5 \times 10^{-7}$; Foxo1 n=322, $p=3.7 \times 10^{-11}$; Tead n=175, not significant (p=0.06); Cebpb n=198, $p=2.1 \times 10^{-3}$; Rbpj n=320, p=0.02; cJun-CRE n=201, not significant (p=0.41); Ets1 n=226, not significant (p=0.06); Stat4 n=385, not significant (p=0.31).

S3N,O - The results shown are representative of 2 independent ChIP experiments for each transcription factor, with similar results.

<u>Fig S4</u>

S4B,C - Number of analysed Zbtb7a-negative promoters n=16789, Zbtb7a-associated promoters with gene expression *not* Zbtb7a-regulated n=639, with Zbtb7a-driven increased expression n=304, with Zbtb7a-driven reduced expression n=273.

S4C - Significances of Zbtb7a-dependent DHS differences at Zbtb7a-negative promoters *vs* promoters with Zbtb7a-driven increased expression: $p=1.3 \times 10^{-56}$, Zbtb7a-driven reduced expression $p=7.3 \times 10^{-72}$ (two-tailed Mann-Whitney u-test; no assumption of equal distribution or variance).

S4F - Number of Zbtb7a-associated promoters with Zbtb7a-dependent gene expression n=629 (total) n=158,156,157,158 (Q1,Q2,Q3,Q4). Significance of difference in Zbtb7a-dependence of DHS between least (Q1) and most (Q4) Zbtb7a-dependent promoters $p=2.3x10^{-14}$; for Zbtb7a-independent promoters *vs* Q1 $p=4.9x10^{-4}$; Q2 $p=4.1x10^{-5}$; Q3 $p=1.4x10^{-11}$; Q4 $p=2.5x10^{-28}$ (two-tailed Mann-Whitney u-test; no assumption of equal distribution or variance).

Fig S5

S5A-G - The results shown are representative of 3 experiments using separate cell lines with independently-restored Zbtb7a expression, with similar results. Error bars indicate s.e.m. of replicate samples within one experiment (n=3). Significances of mRNA expression levels in Zbtb7a-knockout cells with *vs* without restoration of Zbtb7a: *Cd82* $p=9.0x10^{-5}$; *2 Ebf1* $p=1.8x10^{-5}$; *Plau* $p=7.4x10^{-5}$; *Col4a2* $p=4.6x10^{-3}$; *Mustn1* $p=5.2x10^{-4}$; *Postn* $p=1.6x10^{-6}$; *Hp* $p=1.7x10^{-7}$; *Wisp2* $p=2.0x10^{-4}$; *Gsta4* $p=2.6x10^{-4}$; *Glrx* $p=1.9x10^{-4}$; *Xdh* $p=5.8x10^{-5}$; *Ptx3* $p=9.0x10^{-4}$; *Cxcl2* $p=2.1x10^{-2}$; *C3* $p=8.5x10^{-5}$; *Gem* $p=1.1x10^{-3}$ (two-tailed Student's t-test without assumption of equal variance).

Fig S6

S6C,D - DHS-seq using the mutant form of TA3 was performed in 2 separate experiments using independently grown cell cultures, with similar results.

S6E - Significances of induced DHS differences at p65 target promoters +p65 TA3 *vs* +p65 TA3 mutant: $p=7.5x10^{-3}$ (two-tailed Mann-Whitney u-test; no assumption of equal distribution or variance).

S6F - The results shown are representative of 3 independent experiments with similar results; error bars indicate s.e.m of replicate transfections. Significances of TA3-driven reporter expression levels from *Saa3* promoter without mutation *vs* with disrupted Zbtb7a motif: $p=2.5x10^{-2}$ (two-tailed Student's t-test without assumption of equal variance).

S6G - The results shown are representative of 2 independent experiments with similar results; error bars indicate s.e.m of replicate transfections. Significances of *Gem* promoter reporter expression levels in cells co-transfected with Gal4DBD *vs* Gal4DBD-Zbtb7a: p65-knockout cells not significant (p=0.16), +p65 minimal TA3 $p=5.4x10^{-3}$ (two-tailed Student's t-test without assumption of equal variance).
